# Mineralocorticoid receptor antagonism limits experimental choroidal neovascularization and structural changes associated with neovascular age-related macular degeneration

**DOI:** 10.1038/s41467-018-08125-6

**Published:** 2019-01-21

**Authors:** Min Zhao, Irmela Mantel, Emmanuelle Gelize, Xinxin Li, Xiaoyue Xie, Alejandro Arboleda, Marie Seminel, Rinath Levy-Boukris, Marilyn Dernigoghossian, Andrea Prunotto, Charlotte Andrieu-Soler, Carlo Rivolta, Jérémie Canonica, Marie-Christine Naud, Sebastian Lechner, Nicolette Farman, Irene Bravo-Osuna, Rocio Herrero-Vanrell, Frederic Jaisser, Francine Behar-Cohen

**Affiliations:** 1 Inserm UMR_S 1138, Team 17, Centre de Recherche des Cordeliers, 75006 Paris, France; 2 Sorbonne University, University of Pierre et Marie Curie, UMR_S 1138, Centre de Recherche des Cordeliers, 75006 Paris, France; 3 Paris Descartes University, Sorbonne Paris Cité, UMR_S 1138, Centre de Recherche des Cordeliers, 75006 Paris, France; 4 Department of Ophthalmology, University of Lausanne, Jules Gonin Eye Hospital, Fondation Asile des Aveugles, 1004 Lausanne, Switzerland; 5 Ophthalmic Biophysics Center, Department of Ophthalmology, Bascom Palmer Eye Institute, University of Miami Miller School of Medicine, Miami, FL 33136 USA; 6 Department of Computational Biology, Unit of Medical Genetics, University of Lausanne, 1011 Lausanne, Switzerland; 7 IGMM, CNRS, Univ. Montpellier, 34293 Montpellier Cedex 5, France; 8 Department of Genetics and Genome Biology, University of Leicester, Leicester, LE1 7RH UK; 9 Inserm UMR_S 1138, Team 1, Centre de Recherche des Cordeliers, 75006 Paris, France; 10 Department of Pharmacy and Pharmaceutical Technology, Universidad Complutense de Madrid, 28040 Madrid, Spain; 11 Instituto Universitario de Farmacia Industrial, Faculty of Pharmacy, Universidad Complutense de Madrid, 28040 Madrid, Spain; 12 Fundación para la Investigación-HCSC, Instituto de Investigación Sanitaria San Carlos (IdISSC), 28040 Madrid, Spain; 13 Assistance Publique - Hôpitaux de Paris, Hôtel-Dieu de Paris, 75004 Paris, France

## Abstract

Choroidal neovascularization (CNV) is a major cause of visual impairment in patients suffering from wet age-related macular degeneration (AMD), particularly when refractory to intraocular anti-VEGF injections. Here we report that treatment with the oral mineralocorticoid receptor (MR) antagonist spironolactone reduces signs of CNV in patients refractory to anti-VEGF treatment. In animal models of wet AMD, pharmacological inhibition of the MR pathway or endothelial-specific deletion of MR inhibits CNV through VEGF-independent mechanisms, in part through upregulation of the extracellular matrix protein decorin. Intravitreal injections of spironolactone-loaded microspheres and systemic delivery lead to similar reductions in CNV. Together, our work suggests MR inhibition as a novel therapeutic option for wet AMD patients unresponsive to anti-VEGF drugs.

## Abstract (web summary)

Current treatments for age-related neovascular macular degeneration (nAMD) suffer from limited efficacy. Here, Zhao et al. show that pharmacological inhibition or genetic deletion of the mineralocorticoid receptor (MR) limits choroidal neovascularisation in rodents, and show in a pilot clinical study that targeting the MR pathway may provide clinical benefits in nAMD patiens.

## Introduction

Age-related macular degeneration (AMD) is the most frequent cause of blindness in the elderly population in industrialized countries. With a global prevalence of 8%, the projected number of individuals affected in 2020 is 196 million, increasing to 288 million in 2040^1^. Almost a third of early AMD progresses to neovascular AMD (nAMD). Choroidal neovascularization (CNV), in which new vessels growing from the choroid toward the neuroretina underneath the macula, causes macular edema, bleeding, photoreceptors damages, and eventually end stage fibrotic scare (Supplementary Fig. 1). Aging, heredity, diet, smoking, obesity, and vascular diseases are involved in the pathogenesis of AMD^2^; however, the exact mechanisms leading to CNV remain incompletely understood.

CNV is not specific to AMD, it may complicate multiple other diseases affecting the retinal pigment epithelium (RPE) and the choroid, including high myopia and central serous chorioretinopathy (CSCR). The pathogenesis of CNV is complex and multifactorial. Choroidal vessels ensure nutritional and oxygen supply to the avascular outer retina containing the highly energy demanding photoreceptor cells. Choriocapillary loss, observed in nAMD eyes, may cause hypoxia and angiogenesis. A growing body of evidence also indicates that low-grade inflammation, activation of the inflammasome^3,4^, and alternative complement pathway activation play key roles in the pathogenesis of nAMD^5^. In addition to vascular endothelial growth factor (VEGF) family members and their receptors^6^, complement components and pro-inflammatory molecules accumulating in the RPE-choroid complex^7^, such as cytokines and angiopoietins^8^, contribute to CNV growth. The inflammation- and hypoxia-induced downregulation of anti-angiogenic factors such as pigment epithelium-derived factor (PEDF), endostatin, and thrombospondin-1 (TSP-1)^9^ favors a pro-angiogenic microenvironment.

Although multiple molecular pathways have been implicated in the formation and maintenance of CNV, the treatment of nAMD currently relies on biologic compounds that only neutralize VEGF, placental growth factor (PlGF), or both without directly target inflammation^10,11^. Anti-VEGF drugs have strongly improved the visual prognosis of nAMD, allowing the maintenance, and even the restoration of macular function and morphology at the price of multiple intraocular injections^12^. However, anti-VEGFs do not allow CNV regression in nAMD^13,14^. In addition, they have no effect on the fibrotic scarring and may compromise long-term choroid and retinal viability^15,16^. Instead, anti-VEGF agents regulate vascular permeability, as manifested by signs such as edema, which is used to monitor the need for reinjection^17^. In >40% of nAMD cases treated with intensive anti-VEGF treatment for a year, the macula remains wet, suggesting that other pathways are likely to be involved^18^.

Intraocular corticosteroids, a family of potent anti-inflammatory and vasoconstrictor drugs, efficiently reduce macular edema of various origins (diabetic retinopathy, retinal vein occlusion, intraocular inflammation)^19–21^, but show poor efficacy in nAMD. Corticosteroids bind to the glucocorticoid (GR) and mineralocorticoid receptors (MR), both expressed in the retina and choroid^22^. We have previously shown that experimental MR activation mimics CSCR, a retinal disease induced and aggravated by glucocorticoids and associated with subretinal fluid accumulation and frequently complicated by CNV^23,24^. MR antagonists (MRAs) have been shown to be efficient in CSCR^25,26^. In the vasculature, the MR expressed in endothelial and smooth muscle cells contributes to hypertension, vascular inflammation, and fibrosis, for which MRA have beneficial effects^27^. Glucocorticoids are angiostatic^28^, whereas mineralocorticoids have shown both pro- or anti-angiogenic effects depending on the experimental model^29,30^. MRAs showed various anti-angiogenic effects^31^, and spironolactone protected against retinal neovascularization in experimental oxygen-induced retinopathy^32^.

Although a growing body of evidence identifies MR as a player in vascular inflammation, fibrosis, and angiogenesis, their role in the pathogenesis of CNV has not been investigated. In this study, we show that spironolactone, an oral MRA may reduce signs of CNV activity in nAMD patients with resistant active CNV despite monthly intraocular anti-VEGF injections. Using both pharmacologic and transgenic approaches in rodents, we show that antagonism of the mineralocorticoid pathway prevents CNV through a VEGF-independent mechanism. We find that the benefit of spironolactone is additive with anti-VEGF therapy and mediated by the regulation of decorin. These pre-clinical and clinical results identify the MR as a molecular regulatory target for wet AMD.

## Results

### Spironolactone reduces CNV activity in refractory nAMD

**Fig. 1:**
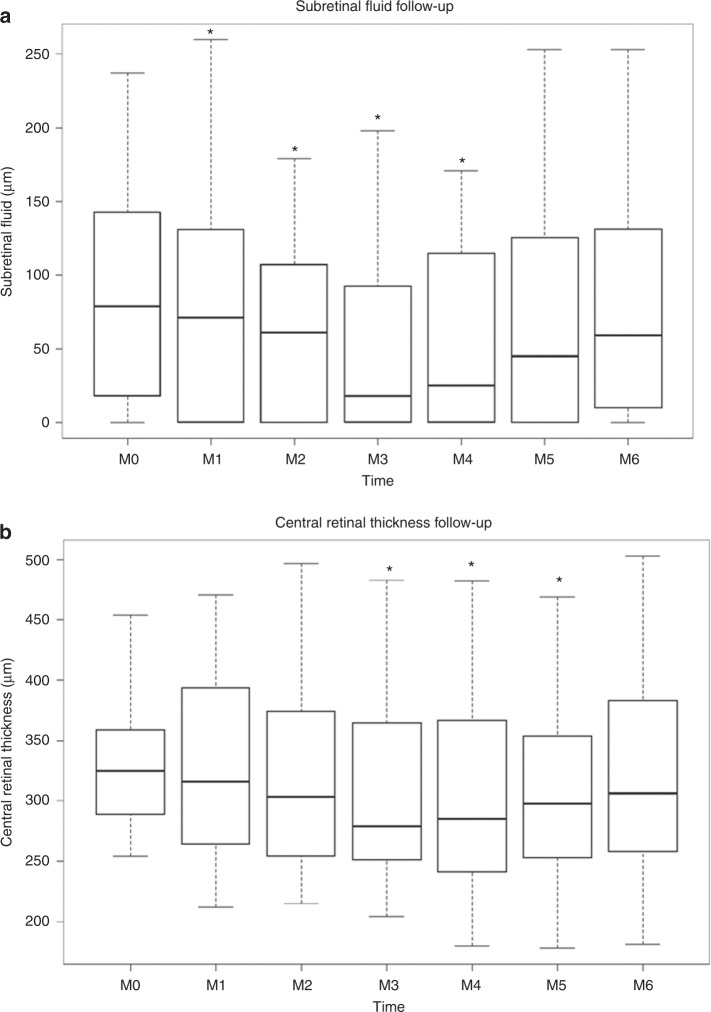
Clinical results. **a** Relative changes in subretinal thickness (SRF, µm) measured with Spectralis OCT during the study period, month 0 (M0) to month 6 (M6). SRF thickness is significantly reduced as compared to M0, at M1 (*p* = 0.012), M2 (*p* = 0.040), M3 (*p* = 0.014), M4 (*p* = 0.024). **b** Relative change in central retinal thickness (CRT, µm) automatic values with Spectralis OCT during the study period, month 0 (M0) to month 6 (M6). CRT is significantly reduced as compared to M0, at M3 (*p* = 0.011), M4 (*p* = 0.007), and M5 (*p* = 0.028). At each time point (Month, M), the data have been summarized using box and whisker plots, the upper and lower 95% CIs (confidence interval) are marked with the whiskers, the box represents the interquartile range, and the median is represented by the bold horizontal line bisecting the box. Paired Wilcoxon signed-rank tests were used. *p < 0.05

Twenty patients with nAMD presenting with refractory intra- or subretinal fluid despite monthly intravitreal injections of anti-VEGF (≥12 months anti-VEGF treatment, ≥6 months refractoriness despite monthly injections, using the same anti-VEGF molecule (Aflibercept in 13 eyes/Ranibizumab in 8 eyes), ≥350 µm on thickest A-scan on optical coherence tomography (OCT)) consented to participate to a prospective pilot study. Refractoriness was defined as no reduction in exudative signs during the last 6 months. In addition to monthly injections of anti-VEGF during the 6-months study period, continuing with the same anti-VEGF molecule as used during the ≥6 months before inclusion, they were prescribed adjuvant oral spironolactone (25 mg/day week 1, 50 mg/day until Month 3, 25 mg/day until Month 4, 0 mg until Month 6). In 21 eyes of 20 patients with refractory nAMD (13 females, mean age 76.3 ± 7.7 (SD) years received 37.2 ± 17.1 (mean ± SD) anti-VEGF injections given over a mean period of 46.0 ± 19.8 (SD) months prior to study enrollment), statistically significant changes in structural outcome measures, maximal at Month 3 compared to baseline, were observed on spectral domain OCT (SD-OCT) at various time points during spironolactone treatment (Fig. 1). The effect was seen in central retinal thickness (*p* = 0.011) and volume (*p* = 0.012), in the foveal thickness (*p* = 0.039), the thickest cystic changes (*p* = 0.040), and subretinal fluid (*p* = 0.014), which all quantify macular edema (Table 1 and Fig. 1, using paired Wilcoxon signed-rank tests). The improvements observed on SD-OCT were lost after stopping the spironolactone at Month 4 although anti-VEGF injections alone were continued (Table 1 and Fig. 1). Individual treatment responses are imaged by thickness maps and a selected B-scan OCT for baseline, 3 and 6 months (horizontal line) for all eyes, obtained with the follow-up mode of the Spectralis OCT (Heidelberg Engineering, Germany) (Supplementary Figs. 2–4). There was a high heterogeneity in the patient’s response to the treatment, with about 50% showing a reduction of fluid at 3 months that could be attributable to spironolactone treatment, which without a control groups remains to be confirmed by a randomized controlled study. Adjuvant oral spironolactone therapy was subjectively well tolerated by most patients. However, two (10%) of them discontinued the drug after 2 months due to increased plasma potassium levels. On the basis of an intention-to-treat approach, these patients were maintained in the analysis.

**Table 1:** Functional and structural outcome measures in nAMD patients treated with spironolactone

	Month 0	Month 1	Month 2	Month 3	Month 4	Month 5	Month 6
Intake of spironolactone before presentation	-	50 mg	50 mg	50 mg	25 mg	-	-
New prescription of spironolactone	25 mg/50 mg	50 mg	50 mg	25 mg	0 mg	0 mg	End of study
Intravitreal anti-VEGF injections	Monthly	Monthly	Monthly	Monthly	Monthly	Monthly	Monthly
Mean maximum retinal thickness [ILM to Bruch’s membrane (µm)]	539 ± 134	523 ± 153 (*p* = 0.10)	517 ± 148 (*p* = 0.08)	484 ± 154 (*p* = 0.042*)	487 ± 151 (*p* = 0.056)	500 ± 153 (*p* = 0.15)	503 ± 158 (*p* = 0.20)
Mean best-corrected visual acuity (ETDRS letters ± SD)	72.8 ± 12.2	72.5 ± 12.4 (*p* = 0.96)	72.0 ± 13.8 (*p* = 0.72)	70.8 ± 13.8 (*p* = 0.48)	71.4 ± 13.2 (*p* = 0.68)	73.0 ± 9.8 (*p* = 0.46)	72.0 ± 11.6 (*p* = 0.96)
Mean central retinal thickness [automatic values from SD-OCT (µm) ± SD]	340 ± 65	332 ± 84 (*p* = 0.38)	326 ± 86 (*p* = 0.32)	311 ± 81 (*p* = 0.011*)	307 ± 84 (*p* = 0.007*)	312 ± 87 (*p* = 0.028*)	322 ± 89 (*p* = 0.18)
Mean central retinal volume [automatic values from SD-OCT (mm^3^) ± SD]	0.268 ± 0.052	0.263 ± 0.066 (*p* = 0.48)	0.255 ± 0.069 (*p* = 0.28)	0.244 ± 0.064 (*p* = 0.012*)	0.242 ± 0.066 (*p* = 0.010*)	0.245 ± 0.068 (*p* = 0.032*)	0.252 ± 0.070 (*p* = 0.18)
Mean foveal retinal thickness [ILM to RPE (µm)]	407 ± 161	384 ± 164 (*p* = 0.063]	377 ± 152 (*p* = 0.039*)	344 ± 148 (*p* = 0.039*)	345 ± 151 (*p* = 0.049*)	354 ± 159 (*p* = 0.088)	355 ± 162 (*p* = 0.099)
Mean of the maximum neuroretinal thickness with cystic changes [ILM to outer segments of photoreceptors (µm)]	329 ± 172	308 ± 166 (*p* = 0.16)	295 ± 160 (*p* = 0.041*)	288 ± 155 (*p* = 0.040*)	293 ± 160 (*p* = 0.010)	304 ± 169 (*p* = 0.19)	300 ± 164 (*p* = 0.17)
Mean subretinal fluid thickness [between outer segment layer and pigment epithelium (µm)]	107 ± 74	86 ± 78 (*p* = 0.012*)	74 ± 58 (*p* = 0.040*)	58 ± 61 (*p* = 0.014*)	70 ± 63 (*p* = 0.024*)	87 ± 80 (*p* = 0.33)	90 ± 75 (*p* = 0.29)
Mean pigment epithelium detachment height [(RPE layer to Bruch’s membrane (µm)]	266 ± 139	266 ± 144 (*p* = 0.93)	266 ± 146 (*p* = 0.87)	244 ± 153 (*p* = 0.27)	245 ± 160 (*p* = 0.34)	248 ± 159 (*p* = 0.40)	254 ± 160 (*p* = 0.54)
Mean subfoveal choroidal thickness [(µm) measured using EDI OCT]	202 ± 87	207 ± 95 (*p* = 0.33)	203 ± 97 (*p* = 0.70)	203 ± 92 (*p* = 0.67)	205 ± 91 (*p* = 0.58)	203 ± 95 (*p* = 0.69)	204 ± 94 (*p* = 0.69)

Patients received spironolactone as add-on treatment to monthly anti-VEGF injections

*SD* standard deviation, *ILM* internal limiting membrane, *RPE* retinal pigment epithelium, *EDI* enhanced depth imaging, *OCT* optical coherence tomography, *SD-OCT* spectral domain OCT, *VEGF* vascular endothelial growth factor

All *p*-values are calculated by comparisons with baseline values using paired Wilcoxon signed-rank tests. *, *p* < 0.05

### Anti-angiogenic effects of MRA in a rat nAMD model

**Fig. 2:**
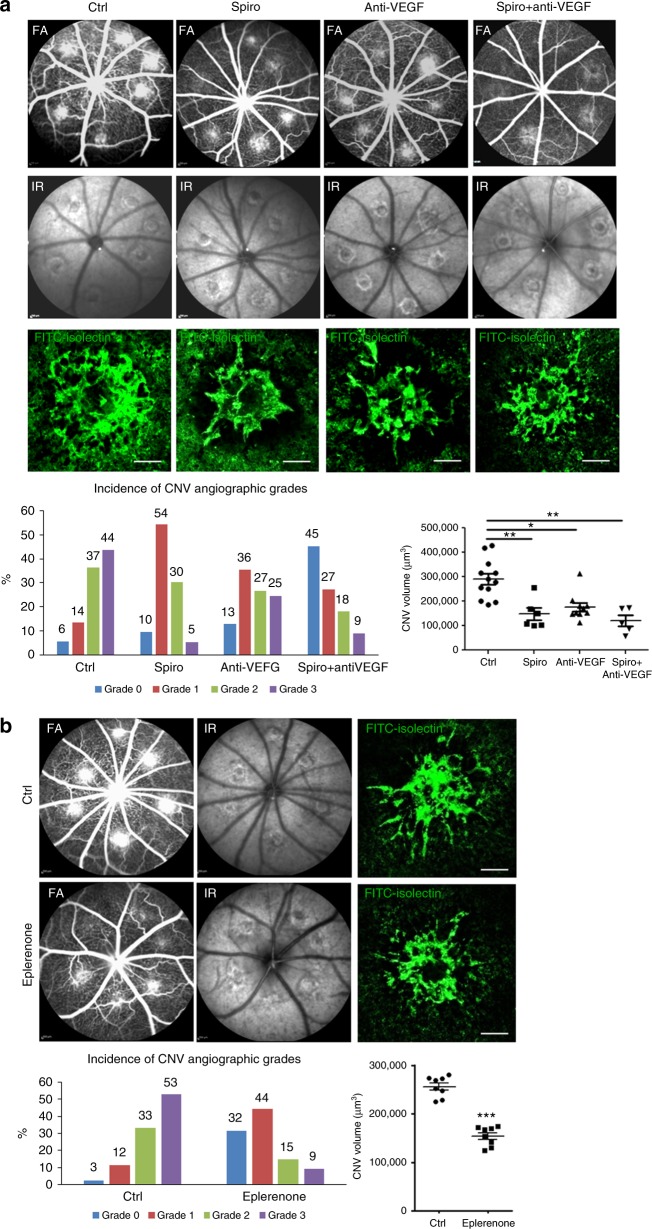
Spironolactone and eplerenone reduce CNV in a rat nAMD model. **a** Spironolactone (Spiro) significantly reduces the CNV angiographic grades evaluated on fluorescein angiography (FA) (*p* = 0.0025) and the CNV volume labeled with FITC-isolectin (green) (*p* = 0.002) as compared to the control group (Ctrl). Infrared images (IR) are used to localize and check the efficient laser-induced burns. The effect of spironolactone is not different from anti-VEGF in reducing the choroidal neovascular leakage on FA and inhibiting the CNV in rat choroidal flat-mounts. Combining the two treatments allows an enhanced effect in reducing vascular permeability compared to anti-VEGF alone (*p* = 0.0335). **b** Eplerenone, a more specific MR antagonist, given orally, significantly reduces CNV angiographic grades (*p* = 0.0012) and CNV volumes (*p* < 0.0001). Bars: 100 µm. FA Data are expressed as the incidence of CNV angiographic grades of the total laser impacts in each group. CNV volumes are expressed as mean ± SEM of average CNV size per rat. *n* represents the number of rats. Linear mixed model was used for statistical analyses. *p < 0.05, **p < 0.01, ***p < 0.001

The above clinical results of anti-edematous effect of MRA might either be linked to an anti-angiogenic effect on CNV or be an anti-edematous effect unrelated to the CNV, as previously described in other retinal conditions^23,26^. To test the anti-angiogenic potential of MRA on CNV, we used an experimental model induced by laser photocoagulation in rodents, a recognized model for nAMD^33^. The argon laser induces a rupture of the Bruch’s membrane, which separates the RPE from the choroidal vasculature, leading to the growth of neovessels from the choroid toward the retina in about 2 weeks. The permeability and size of CNV were assessed in vivo by fluorescein angiography (FA) at day 14 and at day 16 after sacrifice by ex vivo staining with FITC-isolectin on choroid flat-mounts. Spironolactone (25 mg/kg/day, subcutaneous injections from day 0 to day 13) was as efficient as intravitreal anti-VEGF injection (5 µl at 1.5 µg/µl, at day 0) in reducing choroidal vascular leakage assessed by angiographic score and decreasing the volume of the CNV lesions measured on choroid flat-mounts (Fig. 2a). Co-administration of anti-VEGF and spironolactone showed an additive effect on neovascular permeability observed on FA as compared to anti-VEGF alone (Fig. 2a). Systemic spironolactone inhibited the accumulation of allograft inflammatory factor 1 (IBA1)-positive macrophages/microglia in the laser burn area (Supplementary Fig. 5a) and reduced the expression of inflammatory cytokines (monocyte chemoattractant protein 1 [MCP-1], interleukin [IL] 1β, IL6, and tumor necrosis factor [TNF]) in the RPE-choroid complexes at day 3 but had no significant effect on the expression of angiogenic genes such as VEGF, PlGF, ANGPTL4, HIF1α, and TGFβ (Supplementary Fig. 5b). In the rat ocular media, the VEGF level was decreased significantly by anti-VEGF but not by spironolactone; conversely, the MCP-1 level was decreased by spironolactone but not by anti-VEGF (Supplementary Fig. 5c). These results demonstrated the effect of pharmacological MR antagonism on macrophage/monocytes recruitment. Potential non-specific hormonal effects of spironolactone on CNV was excluded as oral eplerenone (INSPRA®, 200 mg/kg/day, 0.2% in chow), a more specific MR antagonist without non-MR related hormonal side-effects^34^ had similar anti-angiogenic effects of rat CNV (Fig. 2b).

To test whether local spironolactone could be as efficient as systemic administration, we elaborated spironolactone-loaded poly(lactic-co-glycolic) acid (PLGA) microspheres(MSs), which can be injected into the vitreous and are well tolerated^35^. The mean size of the particles was 21.87 ± 6.84 (SD) µm. Scanning electron microscopy revealed spherical particles with smooth surface (Supplementary Fig. 6a). The encapsulation efficiency of spironolactone was 77.03% (128.38 µg spironolactone per mg MSs) and a constant release rate of 2.35 µg spironolactone per mg MSs per day was achieved over 31 days (Supplementary Fig. 6a). A single injection (5 µl of 2.2 µg/µl) of spironolactone-loaded MSs in the vitreous of rat eyes at day 0 inhibited choroidal neovascular permeability on FA and significantly reduced the volume of CNV at day 14, as compared to non-loaded-MS-injected eyes (Supplementary Fig. 6b). Therefore, systemic and local spironolactone showed similar anti-angiogenic effects on rat CNV.

### Endothelial MR inactivation reduces CNV in mice

**Fig. 3:**
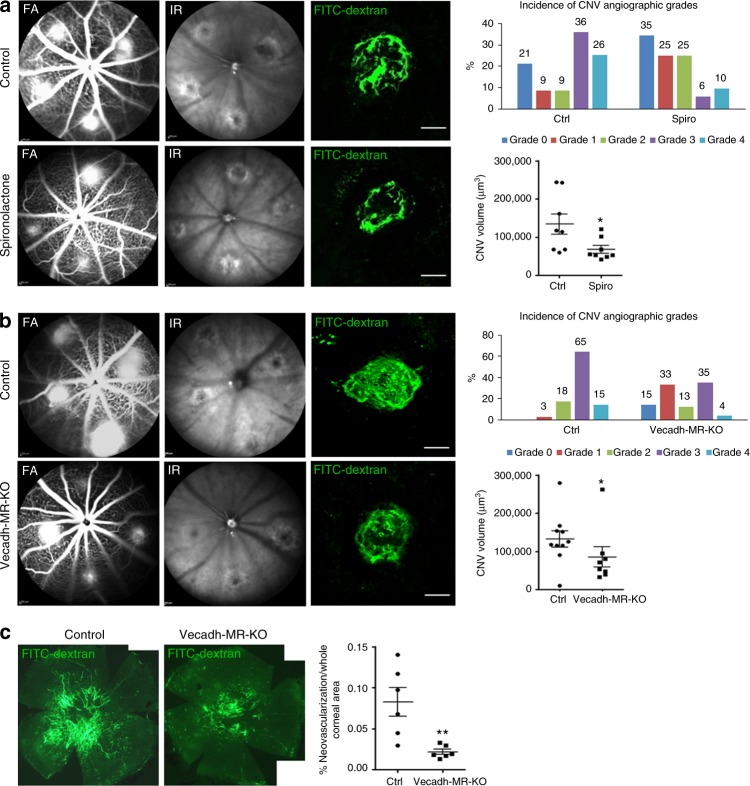
Vascular endothelial MR contributes to CNV. **a** Systemic spironolactone significantly reduces CNV fluorescein angiographic (FA) grades (*p* < 0.001) as well as CNV volume (*p* = 0.0348) as compared to control mice. CNV were labeled with FITC-dextran (green). Infrared (IR) images show up all the laser burns in the fundus. **b** Cell-type-specific MR deletion from endothelial cells using the VE-Cadherin promoter (Vecadh-MR-KO) reduces CNV leakage on FA (*p* = 0.0041) and decreases the volume of CNV labeled with FITC-dextran (green) in mice (*p* = 0.0434). IR images show up all the laser burns. Bar: 100 µm (**a**, **b**). FA Data are expressed as the incidence of CNV angiographic grades of the total laser impacts in each group. CNV volumes are expressed as mean ± SEM of the average CNV size per mouse. *n* represents the number of mice. Linear mixed model was used for statistical analyses. *p < 0.05. **c** In a model of corneal neovascularization using Vecadh-MR-KO mice, a reduction in corneal neovessels labeled with FITC-dextran was observed compared to control mice. Quantification of the neovascular surface on mosaic images confirms a significant decrease in the neovascularization/whole corneal area ratio in Vecadh-MR-KO mice. Data are expressed as mean ± SEM. *n* represents the number of mice. Non-parametric Mann–Whitney *U-*test was used. **p < 0.01

To identify the cellular targets of MRAs, we evaluated the expression and localization of MR in the retinas of rat eyes developing CNV. MR expression was upregulated in the neural retina and in the RPE-choroid complex at day 3 after laser induction, and returned to the basal level at day 16, the late phase of CNV (Supplementary Fig. 7a). Immunohistochemistry showed that the MR was expressed not only in the ganglion cell layer, the inner nuclear layer, the RPE and endothelial cells as described previously^22,23^, but also in the infiltrating cells and in neovascular components within subretinal spaces surrounding the laser burns (Supplementary Fig. 7b–d, arrows). To determine which cell types could drive the MR-induced angiogenic signals, we used conditional transgenic models lacking a functional MR in specific cell types. Specific conditional ablation of MR in endothelial cells only (Vecadh-MR-KO) significantly reduced CNV on FA and on choroidal flat-mounts (Fig. 3b), demonstrating that endothelial cells drive MR-induced angiogenesis. This finding was further confirmed in another ocular model of angiogenesis, in which neovessel growth is induced in the avascular cornea by corneal de-epithelialization. In this model, angiogenesis was also significantly impaired in Vecadh-MR-KO mice (Fig. 3c), suggesting a broader angiogenic effect of the endothelial MR. Of note, targeted MR inactivation in smooth muscle cells (SMA-MR-KO mice) or in granulocytes and macrophages (Lys-MR-KO mice) has no effect on the development of CNV (Supplementary Fig. 8a, b), confirming the endothelial cell specificity of the MR function in angiogenesis. To validate this finding, we used another transgenic model in which MR is constitutively deleted under the endothelial Tie2 promoter (Tie2-MR-Ko), expressed both in endothelial cells and in myeloid cells^36^. Using the laser-induced nAMD model, the angiographic score and the CNV volume were also reduced in Tie2-MR-KO mice (Supplementary Fig. 8c), however, the effect was not significantly different from that observed in Vecadh-MR-KO mice (*p* = 0.1740, repeated measures linear mixed model), suggesting that myeloid MR is not essential in laser-induced CNV. In addition, systemic treatment with spironolactone (Fig. 3a), which antagonizes MR in all cells did not result in a more potent anti-angiogenic effect in mice as compared to the effect observed in Vecadh-MR-KO mice (*p* = 0.5507, repeated measures linear mixed model). Lack of effect on CNV in SMA-MR-KO, which is tamoxifen-inducible, eliminates a potential non-specific effect of Cre with tamoxifen. A non-specific effect of tamoxifen alone was eliminated since control mice received tamoxifen when used to activate the Cre recombinase in the Vecadh-MR-KO mice, and because in the Tie2-MR-KO mice, which is not tamoxifen-inducible, the reduction in CNV was not higher than in the Vecadh-MR-KO mice. Taken together, these results suggest that endothelial MR is required for the development of CNV.

### Decorin mediates angiogenic effect of endothelial MR in CNV

**Fig. 4:**
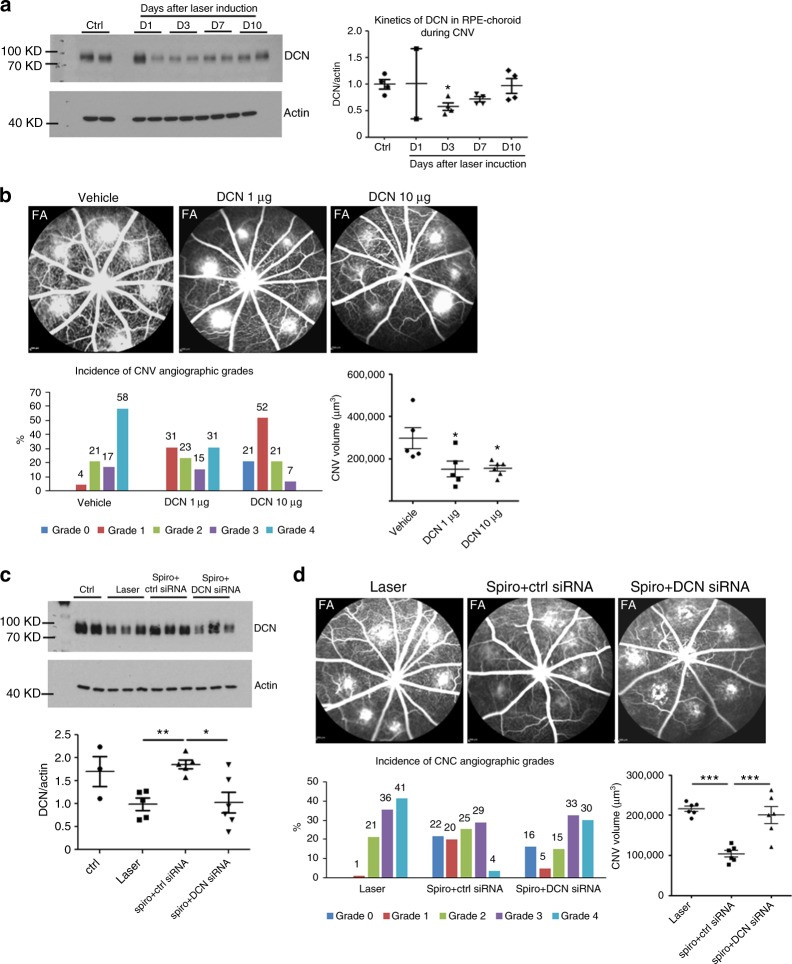
Spironolactone inhibits CNV through induction of anti-angiogenic decorin protein. **a** On western blot, the decorin (DCN) level decreases in the rat retinal pigment epithelium (RPE)-choroid at different time point (day 1, 3, 7, and 10) after laser induction compared to the normal rat RPE-choroid (ctrl). Densitometric quantification shows significant decrease of DCN protein in rat RPE-choroid at day 3 after laser induction. **b** Intravitreal injection (IVT) of recombinant mDCN protein in rat eyes inhibits choroidal vascular leakage on fluorescein angiography (FA); DCN 10 µg/ml significantly reduces the CNV angiographic grades (*p* = 0.02), whereas DCN at both 1 µg/ml (*p* = 0.0461) and 10 µg/ml (*p* = 0.0388) decrease the size of CNV induced by laser. **c** IVT of DCN siRNA in rat eyes with laser-induced CNV. Treatment with spironolactone (Spiro) in the presence of control siRNA significantly increases the DCN protein level in the rat RPE-choroid 48 h after laser induction. IVT of DCN siRNA prevents the increase in the DCN protein induced by spironolactone. **d** Treatment with spironolactone in the presence of control siRNA inhibits choroidal neovascular leakage on FA at day 14 after laser induction (*p* = 0.0194). IVT of DCN siRNA at day 0 and 3 after laser induction abrogates the effect of spironolactone on vascular leakage (*p* = 0.0344). Spironolactone in the presence of control siRNA reduces significantly CNV volume as compared to laser control (*p* < 0.0001). DCN siRNA injected at day 0 and 3 after laser induction abrogates the effect of spironolactone on CNV volume (*p* = 0.0003). Western blot data are expressed as mean ± SEM. Dots represent individual RPE-choroid sample. Non-parametric Kruskal–Wallis test was used. FA Data are expressed as the incidence of CNV angiographic grades of the total laser impacts in each group. CNV volumes are expressed as mean ± SEM of the average CNV size per rat. *n* represents the number of rats. Linear mixed model was used for statistical analyses. *p < 0.05, **p < 0.01, ***p < 0.001

Among the potential molecular targets of the MR in the endothelial cells that could regulate angiogenesis, decorin (DCN) was selected from a full transcriptomic approach designed to identify genes regulated by aldosterone in the whole rat retina consisting of neural retina and RPE-choroid complex (Supplementary data 1). The expression of DCN messenger RNA (mRNA), encoding a matrix protein of the member of the small leucine-rich proteoglycan family, was down-regulated by approximately 2.64 times in the transcriptome of the rat whole retina injected with aldosterone (20 nM final vitreous concentration) compared with that of the vehicle-injected one (Supplementary Data 2). In the rat CNV model, DCN protein expression was reduced in the RPE-choroid as early as 3 days after CNV induction (Fig. 4a) consistent with the timing of MR upregulation in the RPE-choroid (Supplementary Fig. 7a). The DCN levels partly recovered from day 7 to day 10 (Fig. 4a), suggesting that decreased DCN might contribute to the pro-angiogenic balance in this CNV model. To analyze the anti-angiogenic effect of DCN in CNV, we used a recombinant mouse DCN protein that shares 87% amino-acid sequence identity with rat DCN. Intravitreous injection of recombinant mDCN was performed after laser induction at an estimated final concentration of 1 µg/ml or 10 µg/ml in rat vitreous. At day 14, eyes treated with recombinant mDCN at 10 µg/ml showed reduced choroidal vascular leakage (Fig. 4b). The CNV volume was decreased significantly by DCN at both 1 µg/ml and 10 µg/ml (Fig. 4b), confirming a major role of DCN in inhibiting CNV proliferation and leakage. In the rat nAMD model, DCN expression and protein were reduced at day 3 after laser induction and spironolactone upregulated the DCN transcripts (Supplementary Fig. 9a) and the DCN protein levels (Supplementary Fig. 9b) in the rat RPE-choroid, suggesting that the anti-angiogenic effect of spironolactone could be mediated by DCN expression. To test this hypothesis, we designed a strategy to interfere with the effect of spironolactone on DCN expression. We co-administered systemic spironolactone with intraocular siRNA directed against DCN. Spironolactone (in the presence of scrambled siRNAs) significantly prevented the laser-induced decrease in DCN protein at day 2 following induction (Fig. 4c). In contrast, DCN siRNA inhibited the DCN upregulation by spironolactone (Fig. 4c), confirming its efficiency in DCN knockdown. We next tested whether abrogation of the DCN upregulation induced by spironolactone could clinically reduce the anti-angiogenic effect of spironolactone. Rats were treated daily with spironolactone and injected intravitreously with scrambled or DCN siRNA at day 0 and day 3. At day 14, co-administration of spironolactone and DCN siRNA abolished the effect of spironolactone on choroidal neovascular permeability as estimated by FA (Fig. 4d) and reduced the CNV volume (Fig. 4d), demonstrating that DCN mediates, at least in part, the anti-angiogenic effect of spironolactone.

Taken together, these results show that the clinical effect of spironolactone on nAMD refractory to anti-VEGF can be explained by a direct antagonism of the MR in endothelial cells, leading to DCN overexpression.

## Discussion

Anti-VEGF therapy has revolutionized the visual prognosis of CNV, in particular in nAMD patients, from rapid progression to central blindness to central vision stabilized or even improved for several years^12^, demonstrating the key role of VEGF in the regulation of hydro-ionic retinal homeostasis and vascular permeability^37^. However not all nAMD patients respond well to anti-VEGF treatment, particularly when CNV develops underneath the detached retinal pigment epithelium, which represents more than 50% of CNV in AMD cases (Type 1 CNV)^38,39^ and complicates 30–40% of chronic CSCR cases. Moreover, in around 50% of nAMD patients, depending on the drug and regimens, fluid retention persists in the macula despite optimal anti-VEGF treatments^18,40^.  Anti-platelet-derived-growth-factor (PDGF) therapies failed to demonstrate synergistic effects with anti-VEGFs in 2 phase-3 clinical trials^41^, and no other therapeutic options are currently available for nAMD patients not responding optimally to anti-VEGFs. In our clinical pilot study, despite long-lasting anti-VEGF-resistant macular edema, patients treated orally with the MRA spironolactone for 4 months while continuing monthly injection of the same anti-VEGF treatment, showed a significant reduction in signs of CNV activity as measured by FA and SD-OCT. Notably, the effect was lost when spironolactone was stopped, demonstrating that the clinical improvement was an effect of the spironolactone. Despite anatomical improvement, no immediate effect on visual acuity was observed in this group of chronic nAMD patients, which could be due to disease chronicity and irreversible lesions and/ or the short duration of treatment, or sub optimal route of administration. In a recent retrospective study without a control group or controlled time after MRA application, a beneficial effect of MRA on subretinal fluid absorption in anti-VEGF refractory nAMD was also reported^42^. Whether the MRA acted on fluid reabsorption only or also on neovessels could not be determined.

No animal model recapitulates all nAMD features, and the common laboratory animals have no macula. However, laser-induced CNV models in rodents and primate are validated in screening anti-angiogenic drugs for nAMD^33^. In the present study, the MRA spironolactone administrated systemically in rats was as efficient as the reference intravitreal anti-VEGF drug for suppressing CNV. Eplerenone, a more specific MR antagonist, given orally, also efficiently reduced rat CNV, supporting the specific role of MR pathway activation in choroidal neovascularization. Spironolactone significantly inhibited the development of CNV in rats without affecting the ocular VEGF levels; however, MCP-1 expression and microglia/macrophage infiltration at the site of CNV, both known contributors to CNV in AMD^43,44^, were reduced. The monocyte-expressed MR has been reported to stimulate pro-inflammatory macrophages^45,46^. However, MR deletion specifically in myeloid cells did not prevent CNV, and MR deletion in both endothelial cells and myeloid cells did not show a superior inhibitor effect on CNV as compared to MR deletion in endothelial cells only, demonstrating that the benefit of MRA in CNV is not primarily mediated by MR modulation of the macrophage phenotype. In contrast, specific deletion of the MR in endothelial cells significantly inhibited choroidal and corneal neovascularization, indicating that MR activation is pro-angiogenic in wound healing. In addition, systemic spironolactone treatment in mice, which antagonizes all MR expressing cells, did not exert superior anti-angiogenic effect than the deletion of MR specifically in endothelial cells (Vecadh-MR-KO). Similar anti-angiogenic effects of spironolactone have been reported in a model of oxygen-induced retinopathy in which neovessels develops from the retinal vasculature^32^.

The search for molecular pathways regulated by MR potentially modulating angiogenesis and inflammation led us to identify DCN^47^as a potential mediator of the MR effect. In fact, DCN was downregulated in the retina and RPE-choroid complex of aldosterone-injected rats. Furthermore, we showed an anti-angiogenic effect of DCN on CNV. Importantly, laser-induced CNV decreased DCN expression in the RPE-choroid, and this effect was prevented by spironolactone treatment. In addition, a DCN siRNA suppressed the anti-angiogenic effect of spironolactone, identifying DCN as a novel molecular target of spironolactone. In the healthy human eye, DCN is strongly expressed in the cornea, where it intervenes in fibrillogenesis and tissue repair^48^. In the retina, DCN has been identified to be located in all layers but is particularly abundant in Bruch’s membrane, which separates the RPE from the choroid, and in the choroid itself^49^. As a component of the extracellular matrix, DCN can modulate angiogenesis through interaction with numerous molecules^47,50^. In corneal wound healing models and in inflammatory angiogenesis, DCN is anti-angiogenic^51,52^. This effect results from the sequestration of growth factors such as TGF-β, an antagonism of tyrosine kinase family receptors including the VEGF-R2^50,53^, and stimulation of angiostatic molecules such as thrombospondin and tissue inhibitor of metalloproteinase 3 (TIMP3)^54^. Under hypoxic conditions, DCN reduced choroidal endothelial cell proliferation induced by the RPE in culture^55^. In addition, DCN exerts an anti-inflammatory effect through a decrease in macrophage proliferation^56^ and recruitment, in turn mediated by MCP-1 downregulation^57^. In mice CNV, DCN inhibited TGFβ-induced smad2/3 pathway^58^. DCN expression in relation to AMD has not been studied specifically; however, a quantitative proteomic analysis of Bruch’s membrane/choroid from AMD eyes showed significantly decreased DCN levels compared to those in healthy controls^59^. In addition, DCN expression was reduced by approximately 20% in advanced wet as compared to advanced dry AMD eyes^59^, supporting a role for DCN in wet AMD.

In the retina, experimental MR activation has been shown to be pathogenic leading to choroidal vessel dilation, leakage and subretinal fluid retention (mimicking CSCR)^23^, retinal edema mediated by glial hydro-ionic deregulation^22^ and retinal neovascularization in a model of retinopathy of prematurity^32^. In the rodent retina, MR and hydroxysteroid dehydrogenase type 2 are co-expressed in several cell types but not all suggesting that MR pathway could thus be activated by both gluco and mineralocorticoids^23^. Based on clinical observations, MRAs are currently used to reduce edema in CSCR^24,60^. In the present study, we demonstrated that MR activation in vascular endothelial cells contributes to CNV, which is also frequently associated with CSCR. The MR pathway may thus be a common mechanism involved in CNV development in AMD and in CSCR. Why MR is hyper-activated in these conditions is not fully understood. In most organs, the MR are occupied by glucocorticoids, prevalent in the plasma over aldosterone^61^. In nAMD, MR activation may explain the poor efficacy of glucocorticoids on CNV^11^, with their potent anti-inflammatory effects being overridden by the pro-edematous and pro-angiogenic MR pathway. Further studies are required to analyze the MR/GR pathway in AMD.

Local delivery is the method of choice for ocular diseases, allowing higher ocular drug levels without systemic side-effects. For this purpose, slow-release drug delivery systems are mandatory to reduce the frequency of ocular injections. The pre-clinical tolerance of our polymeric spironolactone MS formulation has been demonstrated previously^35^ offering a basis for further clinical testing.

In conclusion, our clinical and experimental data demonstrate that vascular MR activation contributes to CNV. In a pilot study, including patients with refractory and long-lasting exudative signs despite anti-VEGF treatment, we showed that MRA might have an effect on intra and/ or subretinal fluid. In addition, our experimental data support that MR pathway activation contributes to the pathogenesis of nAMD through a VEGF-independent pathway related to DCN, which represents a therapeutic target for nAMD. Further randomized clinical trials, optimally using ocular dedicated formulations, are required to evaluate the therapeutic potential of MR pathway blockade in patients with refractory nAMD.

## Methods

### Patients

A prospective clinical pilot study was performed to evaluate in vivo the effect of spironolactone, an oral MRA, in patients with exudative signs of nAMD. This study was approved by the local ethics committee (#184/14 ethical committee approval, Canton de Vaud), and performed following the regulations of the mentioned committees. It adhered to the Declaration of Helsinki for human research. Informed consent was obtained from all human participants. This study has been retrospectively registered on 16 November, 2018 under the registration number NCT03744767.

Only patients with refractory stable intraretinal or subretinal fluid present for ≥6 months in a row on monthly SD-OCT despite monthly intravitreal of the same anti-VEGF treatment (ranibizumab or aflibercept) were included. The total duration of anti-VEGF treatment before inclusion in the study was required to be ≥12 months to avoid the effect of improvement due to treatment initiation. The thickest macular A-scan from the inner limiting membrane to the RPE band was required to be ≥ 350 µm. Patients giving informed consent were included from September 2014 to March 2015.

The exclusion criteria were polypoidal choroidal vasculopathy, poor imaging quality of the fundus, or any contra-indication to systemic spironolactone (arterial pressure >160/100, K + >5.0 mmol/l, Na + <135 mmol/l, calculated creatinine clearance under 30 ml/min [coefficient × {140-age} × weight/serum creatinine; coefficient = 1.23 for males and 1.04 for females], acute renal failure, renal dialysis, unspecified renal problem, arrhythmia, cardiovascular polymorbidity with thromboembolic risk, known hypersensitivity to spironolactone, ongoing treatment with epleronone, and decompensated hepatic cirrhosis). The treating physician was informed and potentially interacting medications were discussed. During the 6 months study, participants continued fixed monthly intravitreal injections of the same anti-VEGF drug as before, and were prescribed adjunctive oral spironolactone for 4 months (25 mg/day in week 1, then 50 mg/day until Month 3, decreasing to 25 mg/day during Month 4, then stopped) and followed off spironolactone until Month 6. Safety monitoring included history taking for adverse events, arterial blood pressure and laboratory analysis for changes in serum levels of K + , Na + , creatinine, and urea. In case of clinically significant changes, the investigator was allowed to reduce the dosage of spironolactone. At each monthly follow-up visit during the 6 months study, the best-corrected visual acuity (as determined by the Early Treatment Diabetic Retinopathy Study [ETDRS] test score) was recorded and SD-OCT was performed using the follow-up mode. The SD-OCT cubes included 49 horizontal B-scans, encompassing a 6 × 6 mm retinal area, centered on the CNV lesion. The predetermined primary endpoint was the retinal thickness change from baseline to Month 4 (end of adjunctive spironolactone treatment) at the site of maximum retinal thickness (from internal limiting membrane to Bruch’s membrane) measured at baseline and each follow-up visit on the identical A-scan using the inbuilt Spectralis follow-up software. Secondary endpoints included the change in maximum retinal thickness from month 4 to month 6 (anti-VEGF only), and changes in visual acuity (ETDRS letters), automated central retinal thickness (CRT) measurements, automated central retinal volume, the foveal retinal thickness, subretinal fluid, neuroretinal thickness, pigment epithelium detachment height, and choroidal thickness at each monthly evaluation time points.

All quantitative follow-up parameters were statistically analyzed and compared with the baseline parameters using paired Wilcoxon signed-rank tests. For data analysis, Microsoft Excel 2010 and JMP software for Windows (version 8.0.1, SAS Institute Inc., Cary, NC) were used. A two-tailed probability of 0.05 or less was considered statistically significant. The required study sample size was estimated before study initiation. According to the available literature on CSCR, and assuming that the treatment would be less effective in eyes with nAMD, our goal was to examine whether spironolactone could produce a minimum decrease of 15% in all measured retinal thicknesses. Therefore, to achieve a 90% power for detecting this degree of change, we estimated that 21 eyes would be required.

### Microsphere elaboration

PLGA ratio 50:50 (Resomer®503) was purchased from Boehringer Ingelheim Pharma GmbH & Co. (Ingelheim, Germany). Spironolactone was obtained from Sigma-Aldrich (Schnelldorf, Germany). Polyvinyl alcohol 72,000 g/mol (PVA) was obtained from Merck KGaA (Darmstadt, Germany). All organic solvents were high performance liquid chromatography (HPLC) grade and used as received.

Spironolactone-loaded and non-loaded PLGA MSs were elaborated using an oil-in-water (O/W) emulsion solvent evaporation technique^35^. Briefly, The O-phase was prepared by suspension of 40 mg of spironolactone in 1 ml of PLGA solution in methylene chloride (20% w/v) resulting in a Spiro:PLGA ratio of 2:10. This phase was emulsified with the W-phase composed by 5 ml of PVA MilliQ^®^ water solution (2% w/v). The emulsification was performed at 5,000 rpm for 1 min (Polytron^®^ RECO, Kinematica GmbH PT 10–35, Lucerna, Switzerland). This O/W emulsion was subsequently poured onto 100 ml of an aqueous PVA solution (0.1%) and maintained under constant stirring for 3 h to allow MSs hardening. After that, the MSs were washed, filtered, freeze-dried, and kept at −20 °C under dry conditions until used.

### Microsphere characterization

Production yield percentage (PY%) was calculated as the percentage of MS weight divided by the total amount of PLGA and spironolactone initially used in the formulation process.

PY% = (weight of microspheres/initial amount of PLGA and spironolactone) × 100

The mean particle size and particle size distribution were measured in aqueous suspension by light scattering in a Microtrac^®^ S3500 Series Particle Size Analyzer (Montgomeryville, PA, USA).

The external morphology of MSs was evaluated by scanning electron microscopy (Jeol, JSM-6335F, Tokyo, Japan). Particles were gold sputter-coating for observation.

### Spironolactone quantification

For in vitro characterization, spironolactone was quantified by HPLC^35^. Encapsulation efficiency of spironolactone in the microspheres was determined as follows: 5 mg of MSs were dissolved in methylene chloride (1 ml). After that, the polymer was precipitated by addition of 4 ml of ethanol. The polymer suspension was vortex mixing and centrifuged (8500 x *g*; 5 min). The supernatant was recovered and filtered (0.22 µm) for drug quantification by HPLC.

In vitro release studies were performed using 18 samples, which were prepared by suspension of 5 mg spironolactone-loaded MSs in 1.8 ml of release medium (phosphate buffered saline pH 7.4 isotonic with NaCl). Samples were kept at 37 °C under constant agitation at 100 rpm (Clifton Shaking Bath NE5, Nikel Electro Ldt, Avon, UK). At pre-set time point (4, 7, 11, 14, 18, 21, 25, 28, and 31 days) all the samples were centrifuged (8500 x *g*; 3 min; 20 °C). At each time point, two of the pellets obtained after centrifugation were frozen and freeze-dried. The amount of the drug remaining in the particles was quantified from the obtained powders using the same protocol described for encapsulation efficiency and the amount of released drug was calculated as the difference between the initial content and the actual content at each time point. The rest of the supernatants were removed and replaced with the same volume of fresh medium, to continue the release test.

### Animals

All experiments were performed in accordance with the European Communities Council Directive 86/609/EEC and French national regulations and approved by local ethical committees (#2541–2015110210279792 v3, Charles Darwin). Six to eight-week old male Long Evans rats from the Janvier Breeding Center (Le Genest-Saint-Isle, France) were used to create a rat model of CNV. Three-month old male mice with cell-type-specific MR deletion in endothelial, smooth muscle and myeloid cells, (i.e., Vecadh-MR-KO, Tie2-MR-KO, SMA-MR-KO and Lys-MR-KO mouse models, respectively), were generated in the C57BL/6 genetic background as previously described^62–64^. Floxed MR (MRf/f) mice^65^ (kindly provided by Dr. Berger, Heidelberg, Germany) were crossed with mice expressing an inducible Cre-ERT2 recombinase driven by the VE-Cadherin promoter (Cdh5(PAC)-Cre-ERT2 line, kindly provided by Prof. Adams, London, UK;^66^ to generate Vecadh-MR-KO mice) or by the -SMA promoter^67^ (kindly provided by Dr. Metzger, Strasbourg, France; to generating SMA-MR-KO mice). Cell-specific MR inactivation was induced by tamoxifen injection (1 mg/day in corn oil for 5 consecutive days) 14 days before experiments and CNV induction. Both KO and control mice received same tamoxifen regimen.

Lys-MR-KO mice were obtained by mating mice expressing Cre recombinase in myeloid cells (LysMcre;^63^ The Jackson Laboratory, USA) with the floxed MR mice. MRf/f littermates lacking the Cre transgene were used as controls.

Floxed MR mice were crossed with Tie2-Cre mice (strain: B6.Cg-Tg(Tek-cre)12Flv/J, The Jackson Laboratory) to obtain EC MR-/- (MRflox/flox//Tie2-Cre) mice and corresponding MR f/f littermates as controls.

Animals were kept in pathogen-free conditions with food, water and litter, and housed in a 12-h light/12-h dark cycle. Anesthesia was induced by intramuscular ketamine 40 mg/kg and xylazine 4 mg/kg in rats, and intraperitoneal injection of ketamine 50 mg/kg and xylazine10 mg/kg in mice. Animals were sacrificed by carbon dioxide inhalation or cervical dislocation.

### Model of corneal neovascularization

Vecadh-MR-KO mice and their MRf/f littermates were used for the generation of corneal neovascularization. Only the right eye of each animal was used. The corneal epithelium was entirely removed up to the limbus with a surgical microsponge imbibed with 70% alcohol. A suture (7–0 silk) was made to maintain the eyelids closed until day 3. At day 10, mice were perfused with 200 µl FITC-dextran (5 mg/ml, molecular weight 2,000,000, Sigma-Aldrich FD2000S, St-Quentin Fallavier, France) before sacrifice and enucleation. Eyes were then fixed in 4% paraformaldehyde (PFA) for 2 h and washed in phosphate buffered saline (PBS). Corneal buttons were dissected and flat-mounted. FITC-dextran perfused corneal neovessels were observed with a fluorescence microscope (Olympus, Rungis, France). A mosaic of pictures and quantification were obtained using ImageJ. The area of neovascularization was expressed as a percentage of the total corneal area.

### Laser-induced CNV

After anesthesia and dilation of the pupils, coverslips were positioned on the cornea as a contact glass. For rats, six or eight burns (six burns in rat eyes used for fluorescein angiography and CNV quantification; eight burns in rat eyes used for QPCR and western blot analyses) were performed 2 to 3 optic disc diameters away from the optic nerve with an Argon laser (532 nm) mounted on a slit lamp (175 mW, 0.1 s, and 50 µm). For mice, four laser burns were induced at the 3, 6, 9 and 12 o’clock positions around the optic disc (250 mW, 0.05 s, and 50 µm). Both eyes of animals received laser induction. The presence of a bubble witnessed the rupture of Bruch’s membrane and confirmed a successful laser impact.

### Treatments

Treatments were introduced in the rat model of CNV. After laser photocoagulation, rats were divided into eight treatment groups: (1) daily subcutaneous injection of spironolactone diluted in 90% olive oil and 10% dimethyl sulfoxide (DMSO) (25 mg/kg/day) until sacrifice; (2) daily subcutaneous injection of vehicle (olive oil + DMSO) until sacrifice; (3) intravitreous injection (IVT) of anti-rat VEGF (5 µl of 1.5 µg/µl, RnD system, Lille, France) at day 0; (4) co-administration of subcutaneous spironolactone with anti-VEGF IVT; (5) IVT of spironolactone-loaded PLGA MSs at day 0 (5 µl of 2.2 µg/µl); (6) IVT of non-loaded PLGA MSs (5 µl) at day 0; (7) Oral eplerenone (INSPRA®, 200 mg/kg/day, 0.2% in chow) from day 0 until sacrifice; (8) Control normal chow from day 0 until sacrifice. Wild-type C57BL/6 mice were also treated with daily subcutaneous injection of spironolactone (50 mg/kg/day) from day 0 to sacrifice, compared to vehicle injections (olive oil + DMSO).

### Fluorescein angiography (FA)

FA was performed 14 days (in rats) or 10 days (in mice) after laser induction. After pupil dilatation, fluorescein (0.2 ml of 10% fluorescein in saline) was injected intravenously in the tail of rats, or intraperitoneal (0.1 ml) in mice. Early and late phase angiograms were recorded at 1–3 and 5–7 min, respectively, after fluorescein injection. Simultaneously, infrared images were acquired to detect the site and effective presence of laser burn. For each laser-induced lesion, fluorescein leakage was graded qualitatively by evaluating the increase in size/intensity of dye between the early and late phases. Angiographic scores were established by two blinded observers according to the following criteria: grade 0, no hyperfluorescence; grade 1, slight hyperfluorescence with no increase in intensity nor in size; grade 2, hyperfluorescence increasing in intensity but not in size; grade 3, hyperfluorescence increasing both in intensity and size; grade 4, hyperfluorescence size increase more than 2-diameter of the initial laser burn.

### RPE-choroid flat-mounts and CNV quantifications

Two days after FA examination (time necessary for fluorescein elimination), eyes were enucleated, fixed in 4% PFA for 15 min at room temperature and sectioned at the limbus; the cornea and lens were discarded. The retina was separated from the RPE-choroid complex. Eight radial incisions were made on the RPE-choroid, which was then flat-mounted and post-fixed with acetone for 15 min at −20 °C. After washing with 0.1% Triton x100 in PBS, FITC-GSL I-Isolectin B4 (FL-1201, 1:200, Vector, AbCys, Paris, France) was applied overnight at −4 °C. After washing with PBS, the RPE-choroid was flat-mounted and observed with a confocal microscope (Zeiss LSM710, Le Pecq, France). Images of the CNV were captured with a digital video camera coupled to a computer system. Bruch ruptures could be easily observed at each laser spot. Horizontal optical sections (at 1 µm intervals) were obtained from the CNV surface. The deepest focal plane in which the surrounding choroidal vascular network connecting to the lesion could be identified was judged to be the floor of the CNV lesion. The area of CNV-related fluorescence on each horizontal section was measured using the ImageJ software. The summation of the entire fluorescent area on z-stack images from the top to the bottom of the CNV was used as an index for the CNV volume.

Mice were perfused with 200 µl FITC-dextran (FD2000S, 5 mg/ml, molecular weight 2,000,000, Sigma-Aldrich) before enucleation. After RPE-choroidal flat-mounting, the FITC-dextran perfused CNV was examined and analyzed as previously described.

### Immunofluorescence of RPE-choroid flat-mounts

Three days after laser induction (peak of macrophage infiltration), RPE-choroid flat-mounts were also prepared for immunofluorescence. A polyclonal rabbit anti-IBA1 antibody (019–19741, 1:400, Wako, Neuss, Germany) was applied overnight at −4 °C. After washing with 0.1% Triton X100/PBS, the flat-mounts were incubated with AlexaFluo® 594 goat anti-rabbit IgG (A-11012, 1:200, ThermoFisherScientific, Saint Aubin, France), the nuclei were counterstained with DAPI. Images of IBA1 positive macrophage/microglia were observed and captured with a confocal microscope. The IBA1 positive area was measured using the ImageJ software. The average fluorescent area per burn per eye was calculated.

### Immunohistochemistry

Enucleated eyes were fixed in 4% PFA for 2 h, dehydrated and embedded in paraffin. Eight-µm-thick sections were deparaffinized in xylene, hydrated in a graded alcohol series, and washed in PBS-Tween (PBST). After antigen retrieval by heating in citrate buffer and inactivation of endogenous peroxidase by 3% H2O2, sections were incubated with 3% normal horse serum to reduce the non-specific signal. Mouse monoclonal anti-MR 6G1 (1:100, kindly provided by C. Gomez-Sanchez, Division of Endocrinology, University of Mississippi Medical Center, Jackson, MS) was applied overnight at 4 °C. After washing in PBST, sections were incubated with the biotinylated horse anti-mouse IgG BA2000 (1:250, Vector, AbCys, Paris, France) for 45 min at room temperature. Amplification of the signal was obtained with Tyramide Signal Amplification (TSA) kit (Perkin Elmer, Courtaboeuf, France) according to the manufacturer’s instructions. Signal was revealed with 3,3’-diaminobenzidine tetrahydrochloride. Negative controls were performed without a primary antibody.

### Quantitative PCR

For QPCR in rats, eight laser impacts per eye were performed. In all these experiments, *n* corresponds to the number of animals. Three days after laser induction, the rat neuroretinas and RPE-choroid-sclera complexes were carefully dissected from enucleated eyes, snap-frozen in liquid nitrogen and stored at −80 °C until use. Total RNA was isolated from tissues using the RNeasyPlus Mini Kit (Qiagen, Courtaboeuf, France) according to the manufacturer’s instructions. First-strand complementary DNA was synthesized from the total mRNA using random primers (ThermoFisher Scientific) and SuperScript II reverse transcriptase (ThermoFisher Scientific). Transcript levels of MR, MCP-1, IL6, IL1β, TNFα, iNOS, VEGF-A, PlGF, HIF1α, ANGPTL4, TGFβ, and DCN were analyzed by quantitative real-time PCR performed in 7500 Real-Time PCR System (Applied Biosystems, Foster City, CA, USA) with either SYBR Green or TaqMan detection. HPRT1 and 18S were used as housekeeping genes. Delta CT threshold calculation was used for relative quantification of results. Primers are as follows: MR, 5′-TAAGTTTCCCCACGTGGTTC-3′ (forward), 5′-ATCCACGTCTCATGGCTTTC-3′ (reverse); MCP-1, 5′-CCTGCTGCTACTCATTCAC-3′ (forward), 5′-TCT CAC TTG GTT CTG GTC C-3′ (reverse); VEGF-A, 5′-GCGGGCTGCTGCAATG-3′ (forward), 5′-TGCAACGCGAGTCTGTGTTT-3′ (reverse); PlGF, 5′-GTCCTTCTGAGTCGCTGTAG-3′ (forward), 5′-TTCCTCCTTTCTGCCTTTGT-3′ (reverse); HIF1α, 5′-GGTGGATATGTCTGGGTTGAG-3′ (forward), 5′-TTCAACTGGTTTGAGGACAGA-3′ (reverse); ANGPTL4, 5′-TTCTCTACCTGGGACCAAGA-3′ (forward), 5′-CTGTAGTGGATAGTAGCGGC-3′ (reverse); TGFβ, 5′-CAG AAG TTG GCA TGG TAG CC-3′ (forward), 5′-TGC TTC AGC TCC ACA GAG AA-3′ (reverse); DCN, 5′-CTGCTATTCCTCAAGGTCTG-3′ (forward), 5′-AGGAACATTAGCCAGACTGC-3′ (reverse); HPRT1, 5′-GCGAAAGTGGAAAAGCCAAGT-3′ (forward), 5′-GCCACATCAACAGGACTCTTGTAG-3′ (reverse); 18S, 5′-TGCAATTATTCCCCATGAACG-3′ (forward), 5′-GCTTATGACCCGCACTTACTGG-3′ (reverse). IL6,IL1β, TNFα, iNOS were assayed using Thermo Fisher TaqMan (Assays IDs Rn01410330_m1,Rn00676333_g1, Rn01525859_g1,Rn00561646_m1) with HPRT1 as housekeeping gene (Assay ID Rn01527840_m1).

### IVT of recombinant DCN protein

We used recombinant mouse DCN protein (R&D Systems) that shares 87% amino-acid sequence identity with rat DCN. IVT of DCN was performed after laser induction at a final concentration of 1 µg/ml or 10 µg/ml in the rat vitreous. FA was performed at day 14, and CNV quantification on choroidal flat-mounts at day 16. Rat was chosen for this experiment as CNV is more reproducibly induced in rats than in mice, the eye size is larger allowing collection of more material and repeated injections are easier to perform in the larger rat eye.

### DCN silencing by siRNA in CNV model

Rat DCN stealth siRNAs (set of 3, RSS307035, RSS307036, RSS307037) were purchased from ThermoFisher Scientific. Stock solution was made by adding 1 ml H_2_O to 20 nMol DCN siRNA set. Before IVT, for each rat, siRNA injection solution was prepared by mixing 2 µl siRNA stock solution with 10 µl Mirus transfection reagent and vortexed. After laser induction, 5 µl of siRNA solution was injected in each eye of a rat. Control rat eyes were injected with scrambled siRNA (Stealth™ RNAi negative control, medium GC duplex, ThermoFischer Scientific) prepared according to manufacturer’s instruction. Animals were sacrificed at day 2 after laser induction, and the RPE-choroid complexes were retrieved for western blotting. To test the effect of DCN silencing on CNV formation, DCN siRNA was injected intravitreously in rat eyes at day 0 and day 3 after laser induction. FA was then performed at day 14 to evaluate the permeability of choroidal neovessels. CNV was stained using FITC-GSL I-Isolectin B4 on RPE-choroid flat-mounts and quantified using Image-J.

### Western blotting

For protein analysis, we performed eight laser impacts per rat eye. The experiment was repeated twice independently. The dissected RPE-choroidal samples were processed using standard methods^23^ for western blotting. Briefly, equal amounts of protein (20 µg) were separated on Novex® 4–20% Tris-Glycine gel (Thermo Fisher Scientific), transferred to nitrocellulose, and the blots were incubated with rabbit anti-DCN (ab175404, 1:1000, Abcam, Cambridge, UK) at 4 °C overnight. The membranes were then washed, incubated with HRP goat anti-rabbit IgG antibody (PI-1000, 1:5000, vector) for 1 h at room temperature, and developed using ECL Plus western blotting detection reagents (GE healthcare, Orsay, France). Scans of blots were provided as Supplementary Fig. 10. The goat anti-Actin (sc1616, 1:2500, Santa Cruz Biotechnology, Heidelberg, Germany) was used as internal control.

### Statistical analysis

In rat and mouse CNV models, in order to take into account simultaneously the correlation between the two eyes of an animal and the correlation for repeated measurements in the same eye (in case of repeated impacts), a linear mixed model for repeated measures LMMRM (known to be robust to the normality assumption) was used, including treatment (or mutation), side, time and treatment х time as fixed effects and animal as a random effect. No adjustment for multiplicity was used at this level, knowing the exploratory purpose of the pre-clinical data.

When pooling different experiments for comparison purposes, a specific model (LMMRM) on control animals only was performed to check the comparability and the lack of heterogeneity between experiments. As no significant difference was shown between controls (no experiment effect), the pooling was considered interpretable.

Analyses were performed in SAS® 9.4 for Windows, SAS Institute Inc., Cary, NC.

For data related to QPCR and western blot, comparison between two groups was performed using Mann–Whitney *U*-test. Comparison between multiple groups was analyzed using non-parametric Kruskal–Wallis followed by Dunns test (GraphPad Prism 5 for Windows, GraphPad Software Inc., San Diego, CA, USA). *p*-values of 0.05 or less were considered significant.

### RNA-sequencing and data analysis

Male 8-week-old Lewis rats were injected intravitreously with aldosterone (20 nM final concentration in the vitreous) and sacrificed after 24 h. Control rats received vehicle injection. The whole retinas (comprising neuroretina and RPE-choroid complex) were isolated for RNA-sequencing. Total RNA was extracted using the RNeasyMini Kit (Qiagen) including DNase I treatment. RNA integrity was checked on the Agilent 2100 Bioanalyzer. At least three independent biological replicate samples were sequenced and used for downstream analysis. RNA-sequencing was performed on Illumina HiSeq2000 platform. The average number of reads per sample was 27 M. Reads from each sample were processed as follows. First, reads were trimmed using an in-house Perl script with a minimum phred quality of 20 per vase and a minimum read length of 30 bp. On average, 24% of reads per sample were discarded. The resulting reads were later aligned to the Rattus Norvegicus genome assembly 3.4 (from Ensembl) using Tophat version 2.0.10. At least 12 million reads were aligned to the genome for each sample. We next quantified gene expression to obtain read count and FPKM values. The non-adjusted read counts for each gene were used for statistical calculation of global differential expression using DESeq2. Differentially expressed genes were selected at an adjusted *p*-values of ≤ 0.05 and fold changes >1.5.

Differential expression between aldosterone- and vehicle-injected rats was also analyzed using the GOlandscape, a threshold-free gene ontology tool (The GOlanscape R-package and a detailed description of the tool are available at the website https://github.com/andreaprunotto/GOlandscape). This software computes a stepwise GO-analysis all over the range of DE significance, assigning a *p*-value for each GO term at each step. Then it selects the most significant annotated categories in the range, displaying them in combination with the most represented annotated genes within these terms. The output is a heatmap, which enables the analyst to identify quickly which gene is involved in which process (at the maximum level of statistical significance), independently on an arbitrary choice of the DE genes list.

### Code availability

The R code of GOlandscape used in this study and related documentation are available at the websites https://github.com/andreaprunotto/GOlandscape and https://zenodo.org/record/1474122.

### Reporting Summary

Further information on experimental design is available in the Nature Research Reporting Summary linked to this Article.

## Supplementary Information

Supplementary Information

Description of Additional Supplementary Files

Supplementary Data 1

Supplementary Data 2

Reporting Summary

## Data Availability

All relevant data are available from the corresponding author upon reasonable request. RNA-sequencing data are available from ArrayExpress database at EMBL-EBI under accession number E-MTAB-7438. The images from figures are available at figshare. A Reporting Summary for this Article is available as a Supplementary Information file.
