# Supplementary material for: Mineralocorticoid receptor antagonism limits experimental choroidal neovascularization and structural changes associated with neovascular age-related macular degeneration
Source: Nat Commun. 2019 Jan 21;10:369. doi: 10.1038/s41467-018-08125-6 (PMC6341116; doi:10.1038/s41467-018-08125-6)
Supplement: Supplementary file 1 — Supplementary Information [file 41467_2018_8125_MOESM1_ESM.pdf]

Supplementary information

Mineralocorticoid receptor antagonism limits experimental choroidal neovascularization and structural changes associated with neovascular age-related macular degeneration

Zhao et al.

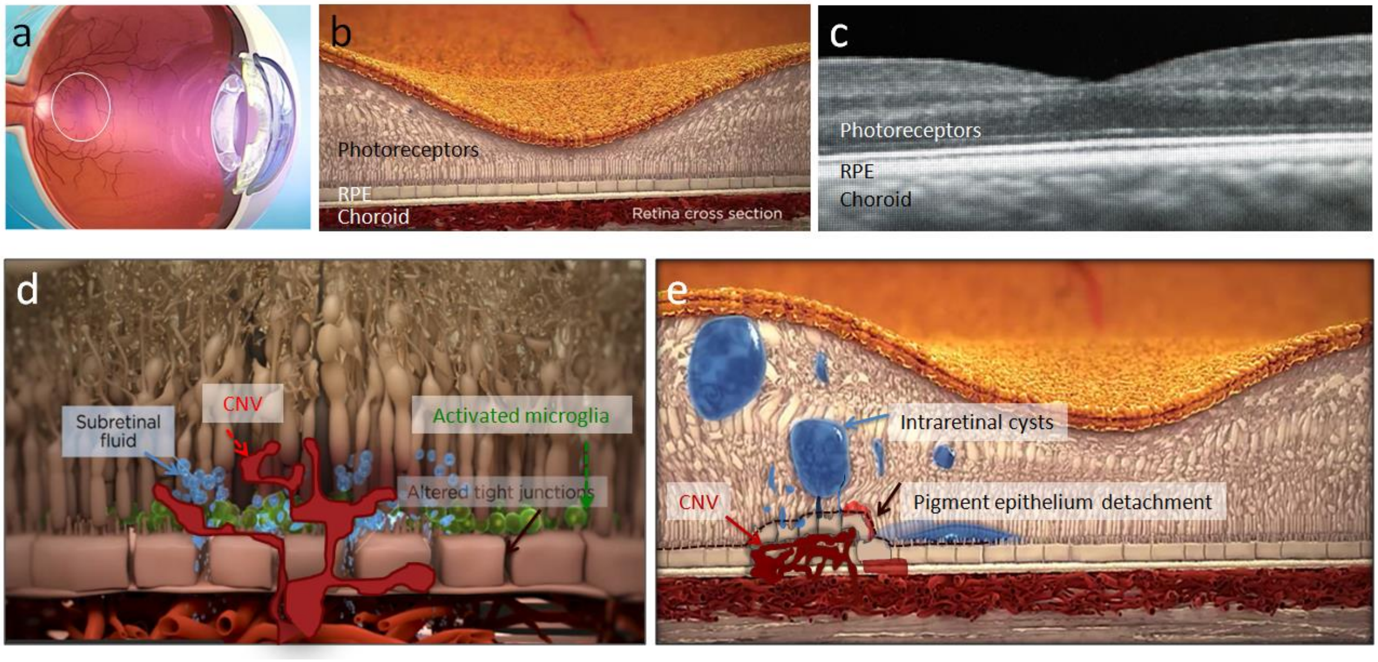

Supplementary Figure 1. Schematics of CNV leading to intra- and subretinal fluid on human macula cross section.

(a) Location of the macula (circle) in the posterior part of the retina observed from a schematic longitudinal section of the ocular globe. Cross-section of a human macula in a schematic representation (b) and on SD-OCT (c). Representation of CNV growing from the choroid through the retinal pigment epithelium (RPE) under the retina (d) or under a detachment of the RPE, causing accumulation of fluid under the retina and in intraretinal cysts (e).

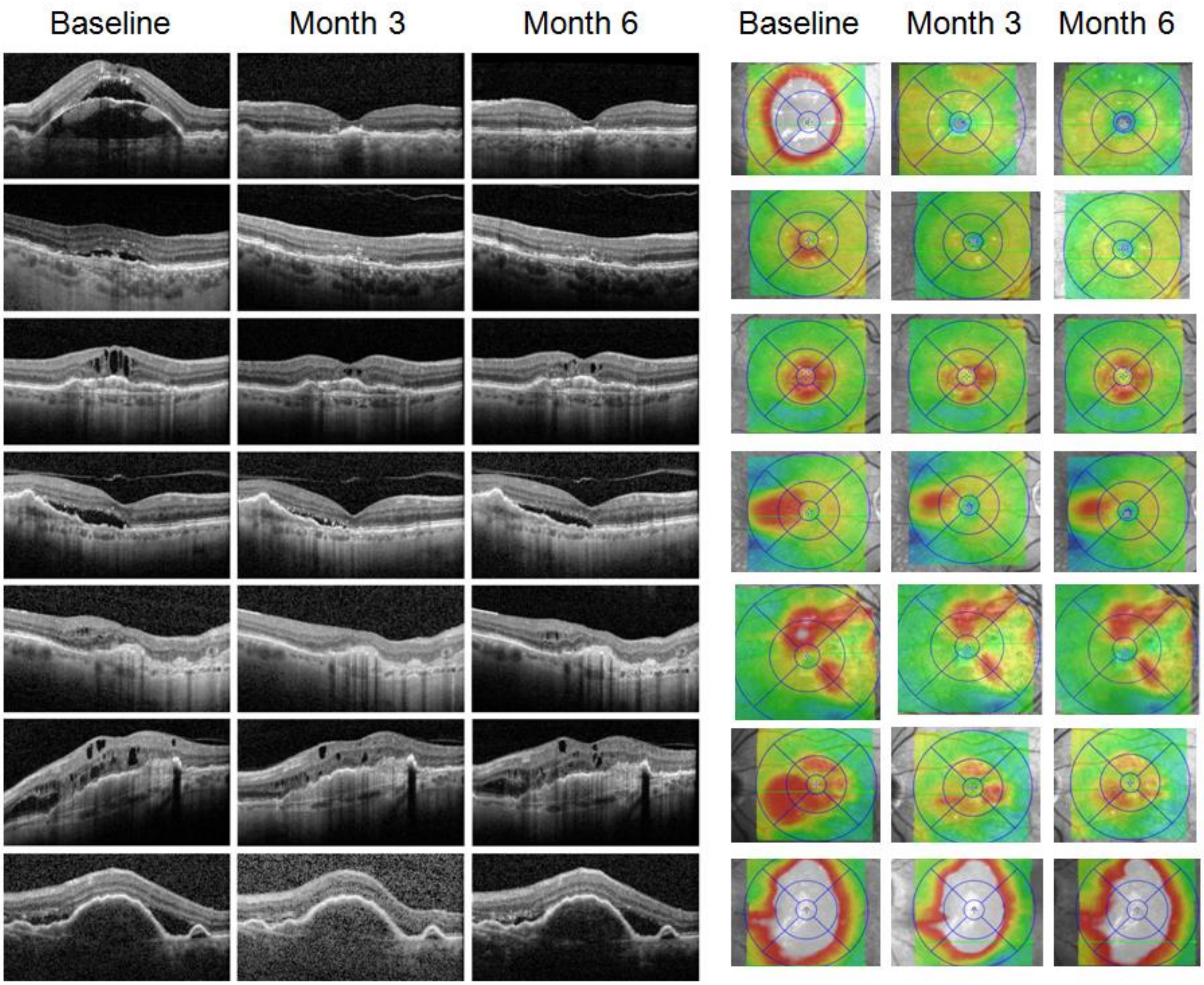

Supplementary Figure 2. SD-OCT B-scan and thickness map for nAMD eyes 1-7.

Pictures of patients at baseline, month 3 and month 6.

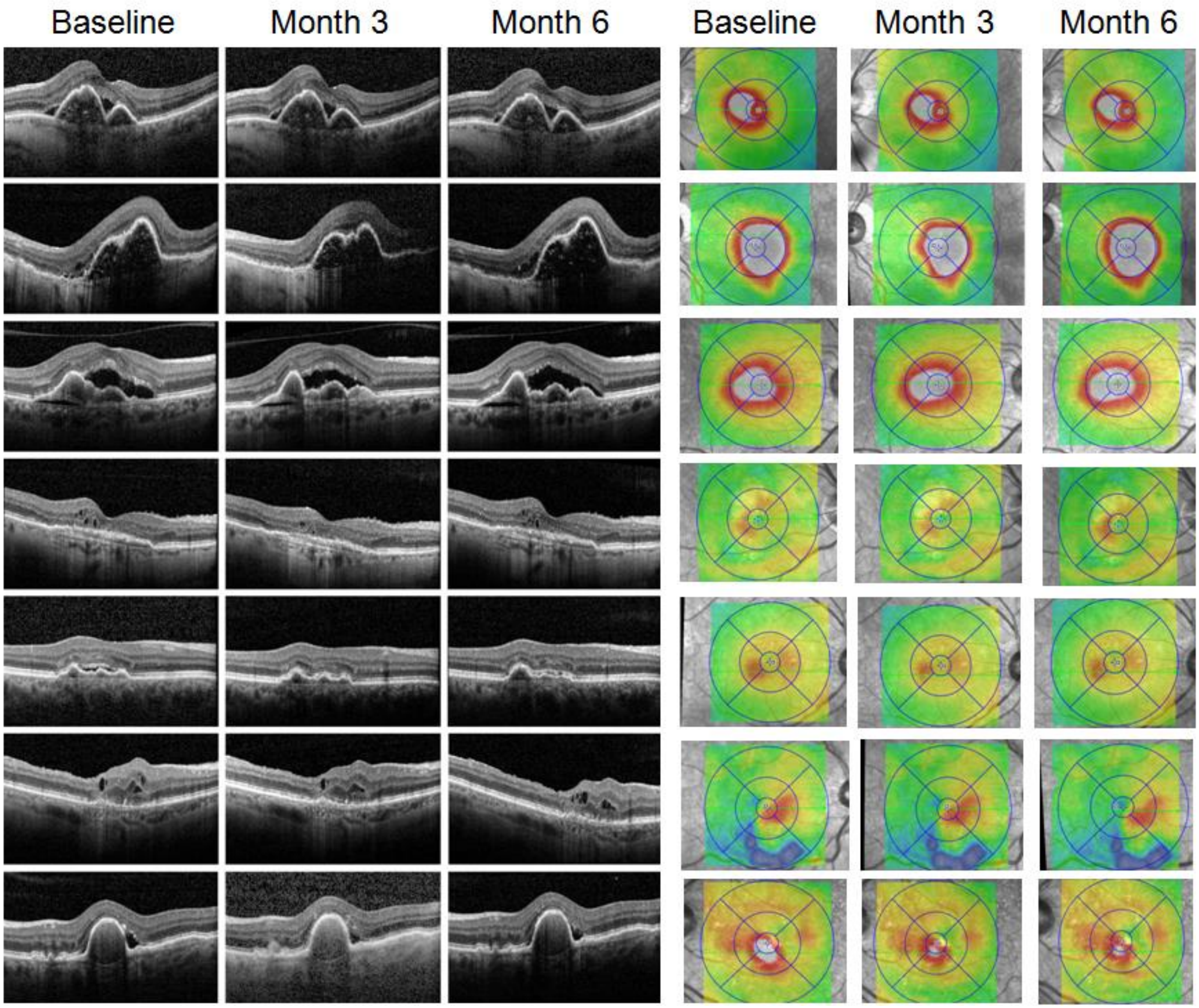

Supplementary Figure 3. SD-OCT B-scan and thickness map for nAMD eyes 8-14.

Pictures of patients at baseline, month 3 and month 6.

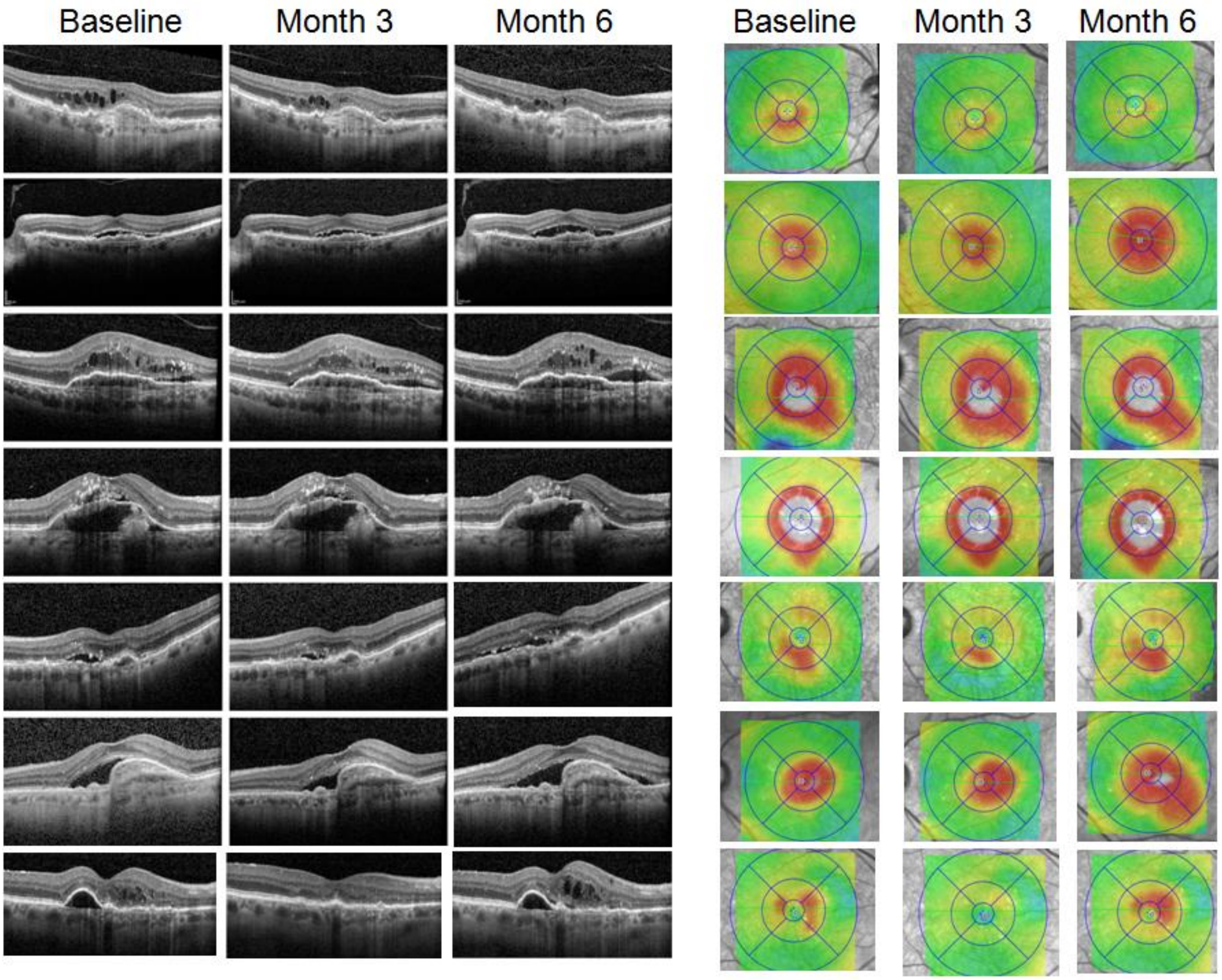

Supplementary Figure 4. SD-OCT B-scan and thickness map for nAMD eyes 15-21.

Pictures of patients at baseline, month 3 and month 6.

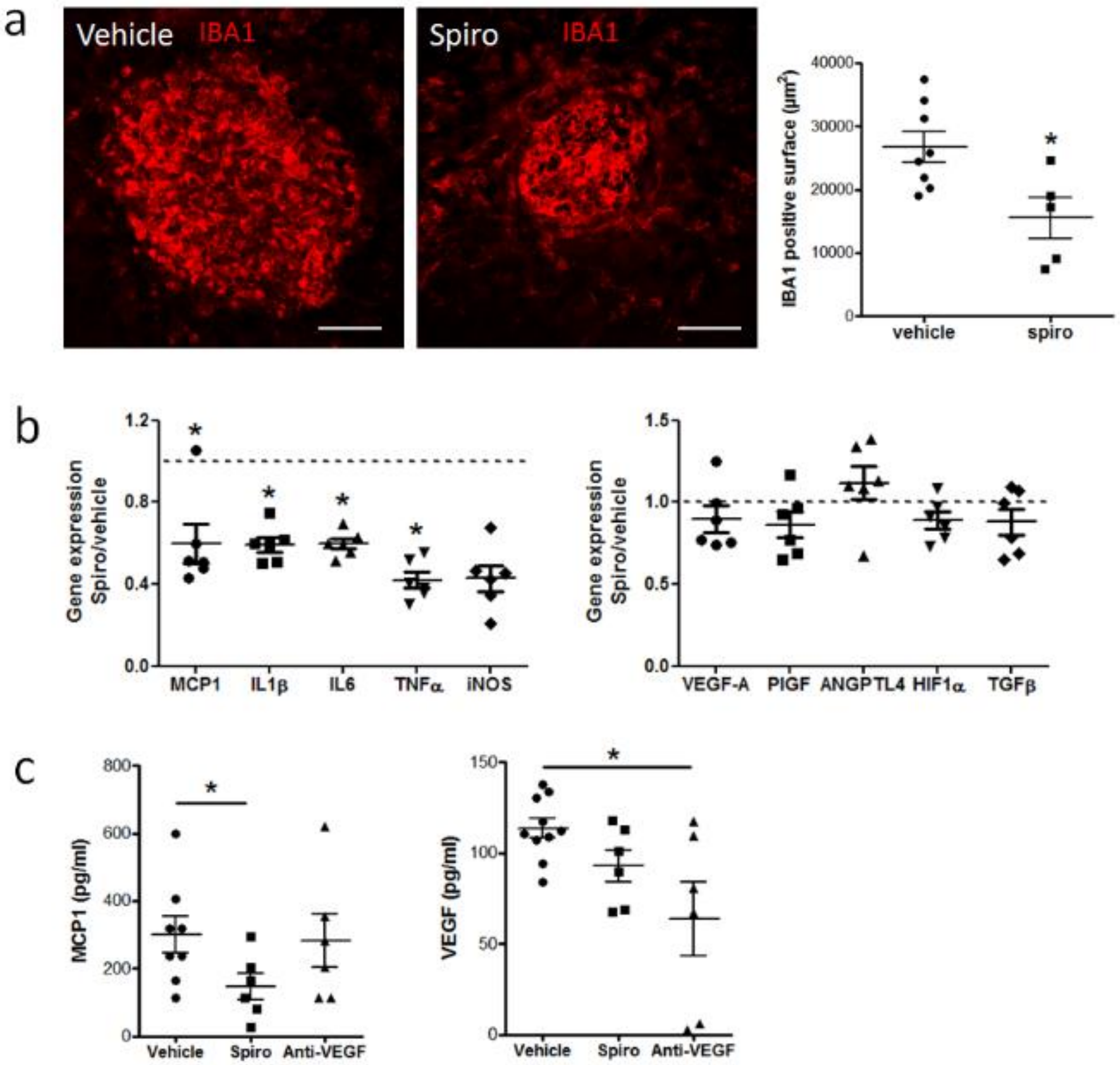

Supplementary Figure 5. Spironolactone inhibits inflammation in rat RPE-choroid induced by laser.

(a) Spironolactone inhibits the accumulation of IBA-1-immunolabelled macrophages/microglia (red) in the areas of laser burns on rat choroidal flat-mounts. Bars: 100 µm.

(b) Quantitative PCR of rat RPE-choroids at day 3 after laser induction shows spironolactone-induces down-regulation of the expression of pro-inflammatory genes (MCP1, IL1B, IL6 and TNFα) compared to vehicle-injected laser controls (dashed line). However, the expression of VEGF-A, PlGF, ANGPTL4, HIF1α and TGFβ does not change.

(c) ELISA assay performed on rat intraocular liquid collected at day 3 after laser induction shows that spironolactone reduces the concentration of MCP1 but does not have effect on VEGF. In contrast, anti-VEGF inhibits intraocular VEGF but does not reduce the induction of MCP1 in laser-induced CNV.

Data are expressed as mean ± SEM. n represents number of rats. Non-parametric Mann-Whitney test (2 groups) or Kruskal-Wallis test (3 groups) was used. *, $p < 0.05$.

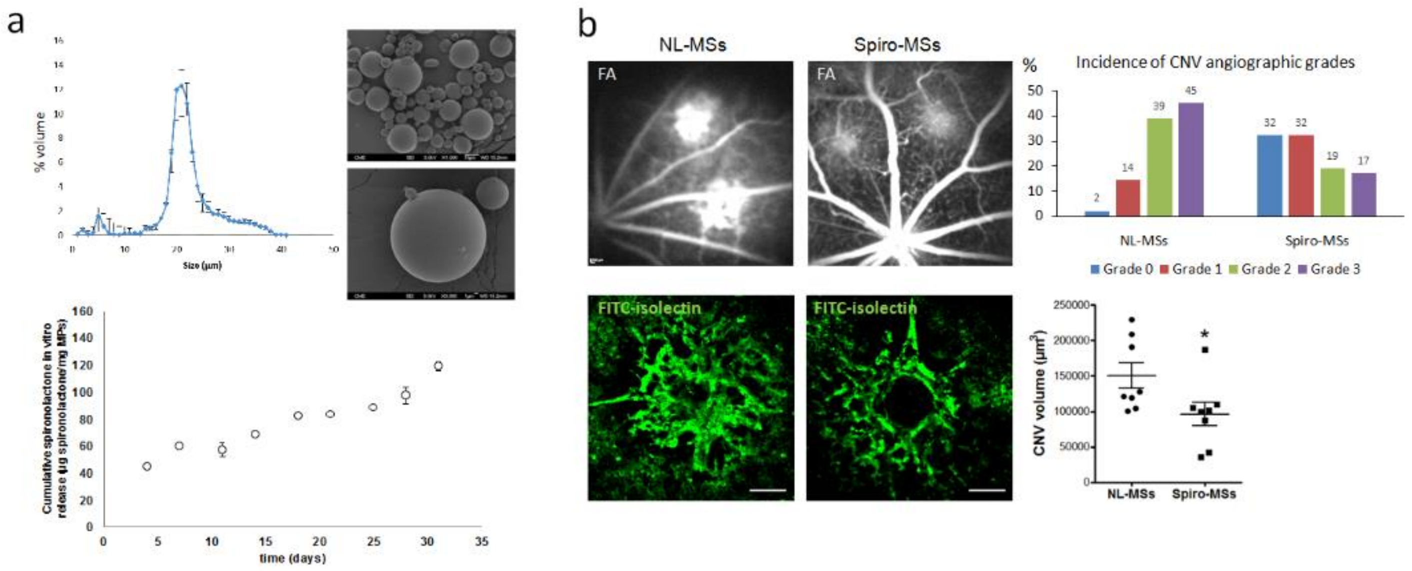

Supplementary Figure 6. Effect of intravitreal spironolactone-loaded PLGA microspheres on rat CNV.

(a) Particle size distribution of spironolactone-loaded PLGA microspheres (Spiro-MSs), scanning electron microscopy images of MSs (upper), and cumulative *in vitro* release of spironolactone (µg/mg MSs) over 31 days from spironolactone-loaded PLGA MSs in PBS (pH7.4) (lower).

(b) Spiro-MSs injected in the vitreous of rat eyes inhibit the vascular leakage on fluorescein angiography (FA) compared to non-loaded MSs (NL-MSs) (upper) ($p = 0.0391$). CNV labeled with FITC-isolectin (green) shows a significantly reduced size in rat eyes injected with spiro-MSs compared to those injected with NL-MSs (lower) ($p = 0.0207$). Bar: 100 µm. FA Data are expressed as the incidence of CNV angiographic grades of the total laser impacts in each group. CNV volumes are expressed as mean ± SEM of the average CNV size per rat. n represents the number of rats. Linear mixed model was used for statistical analyses. *, $p < 0.05$.

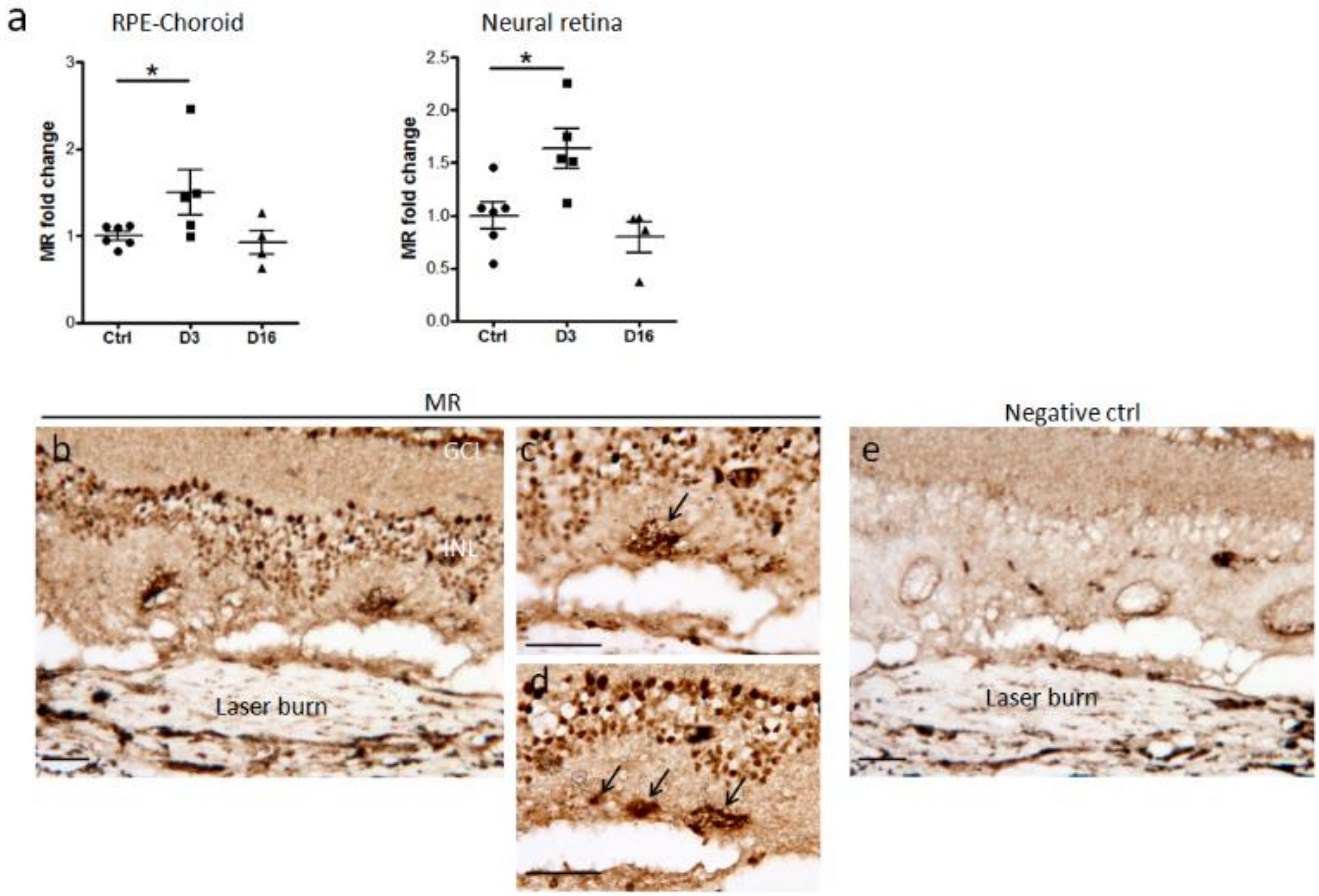

Supplementary Figure 7. Expression and immunolocalization of MR in the retina of rat nAMD model.

(a) MR expression is up-regulated in rat RPE-choroid and neural retina at day 3 (D3) after laser induction, while at day 16 (D16), there is no difference in MR gene expression compared to the control rats (ctrl). Data are expressed as mean ± SEM. n represents the number of rats. Non-parametric Kruskal-Wallis test was used. *, $p < 0.05$.

(b-d) MR is expressed in the cells in the inner nuclear layer (INL), and cells accumulated in the subretinal space (arrows) around the laser burned area. (e) shows the negative control. Bar: 100 µm.

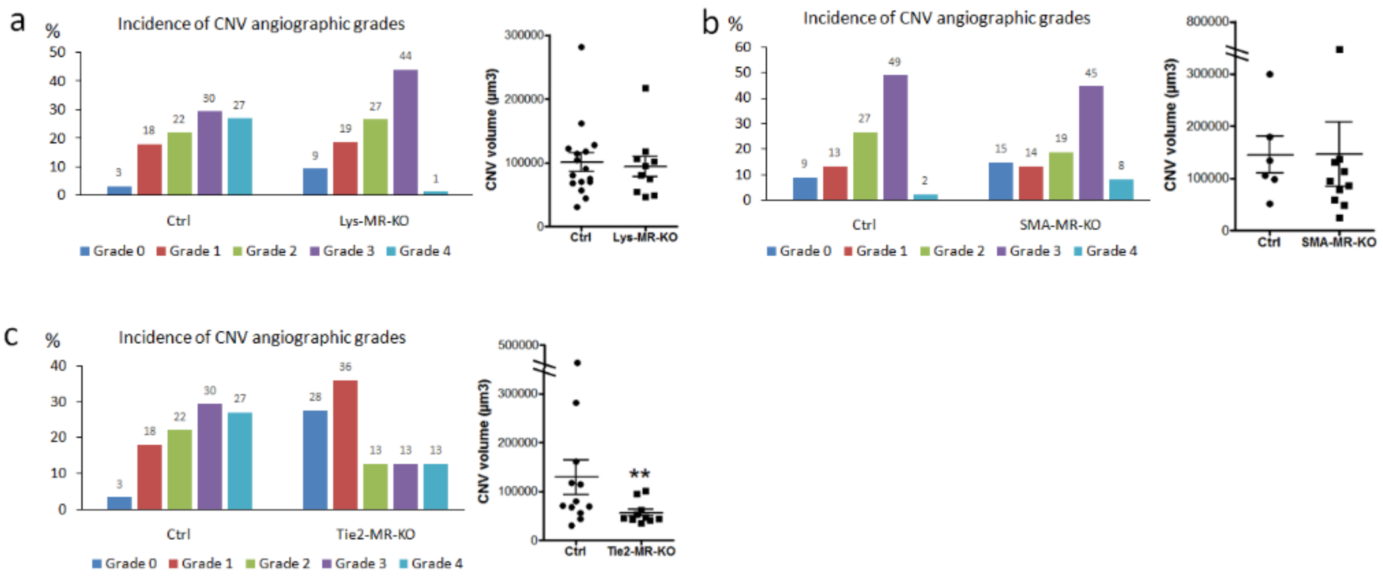

Supplementary Figure 8. Effect of cell-type-specific MR deletion on CNV.

Cell-type-specific MR deletion in myeloid (constitutive Lys-MR-KO, a) or smooth muscle cells (tamoxifen-induced SMA-MR-KO, b) does not prevent CNV in mice, as there is no difference in the angiographic grades and CNV volume compared to control mice. Constitutive MR deletion in Tie2 expressing cells (endothelial and myeloid cells) (Tie2-MR-KO, c) reduces CNV angiographic scores ($p = 0.0066$) and CNV volumes ($p = 0.0059$). FA Data are expressed as the incidence of CNV angiographic grades of the total laser impacts in each group. CNV volumes are expressed as mean ± SEM of the average CNV size per mouse. n represents the number of mice. Linear mixed model was used for statistical analyses. **, $p < 0.01$.

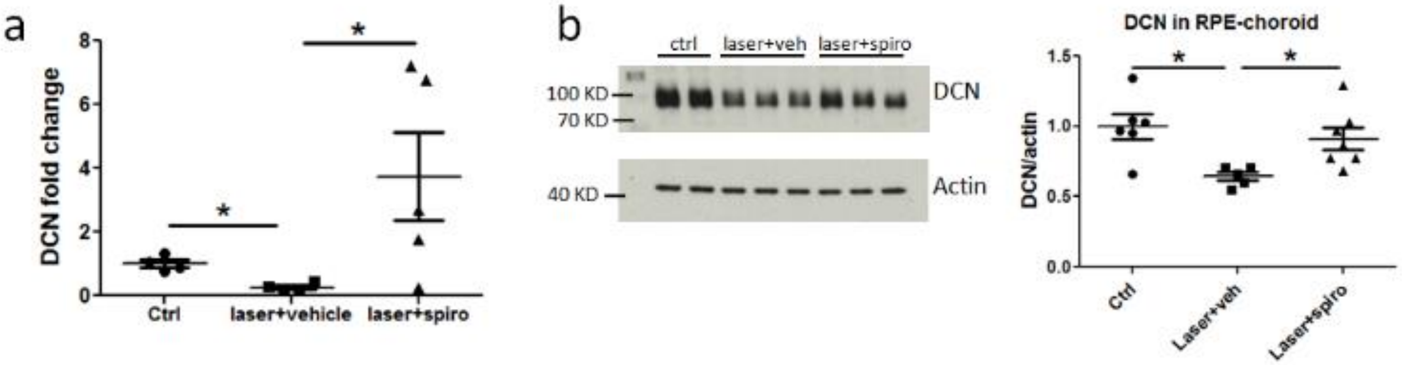

Supplementary Figure 9. Spironolactone increases decorin in rat RPE-choroid after laser.

(a) Quantitative PCR shows a down-regulation of the transcripts of decorin (DCN) in the RPE-choroid at day 3 after laser induction and vehicle treatment, and an up-regulation of DCN after spironolactone (spiro) treatment. (b) Western-blot shows a reduction of DCN protein in the RPE-choroid at day 3 after laser induction and vehicle treatment and an increase in DCN after spironolactone treatment. Data are expressed as mean ± SEM. n represents the number of rats. Non-parametric Kruskal-Wallis test was used. *, $p < 0.05$.

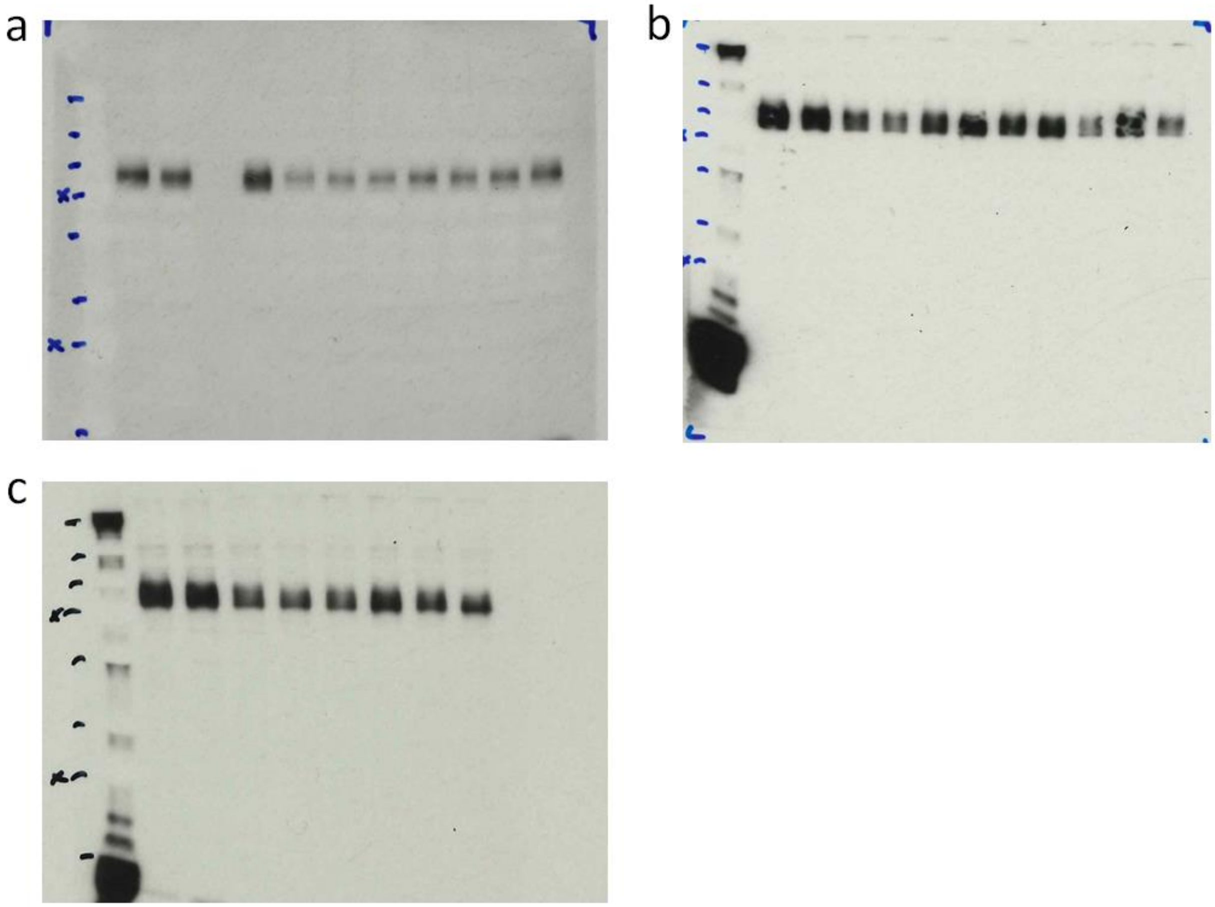

Supplementary Figure 10. Scans of whole western blot membranes of decorin.

(a) in Figure 4a, (b) in Figure 4c and (c) in Supplementary Figure 9b.
