# Supplementary material for: Mineralocorticoid receptor antagonism limits experimental choroidal neovascularization and structural changes associated with neovascular age-related macular degeneration
Source: Nat Commun. 2019 Jan 21;10:369. doi: 10.1038/s41467-018-08125-6 (PMC6341116; doi:10.1038/s41467-018-08125-6)
Supplement: Supplementary file 2 — Description of Additional Supplementary Files [file 41467_2018_8125_MOESM2_ESM.docx]

**Description of Additional Supplementary Files**

**File Name:** Supplementary data 1.

**Description:** GOlanscape output related to ALDO-RET comparison (pooling together up- and downregulated genes). The heatmap displays the first 30 Molecular-Function GO-entries (rows) in relation with the first 80 most differentially expressed genes (columns). The rank of the GO-entries (top, most significant) and genes (right, most significant) is the global significance, which combines differential expression p-values and GO-p values along their full ranges.

**File Name:** Supplementary data 2

**Description:** Genes regulated by aldosterone in the whole retina of treated rats. List of genes differentially expressed in the retinas of aldosterone versus control treated rats. The presented genes were selected based on log fold change values logFC (Control/Aldo) >0.74 and statistical significance (P-value ≤ 0.05). Genes were sorted by log fold change values.
