# Supplementary figures and images for: Mineralocorticoid receptor antagonism limits experimental choroidal neovascularization and structural changes associated with neovascular age-related macular degeneration
Source: Nat Commun. 2019 Jan 21;10:369. doi: 10.1038/s41467-018-08125-6 (PMC6341116; doi:10.1038/s41467-018-08125-6)

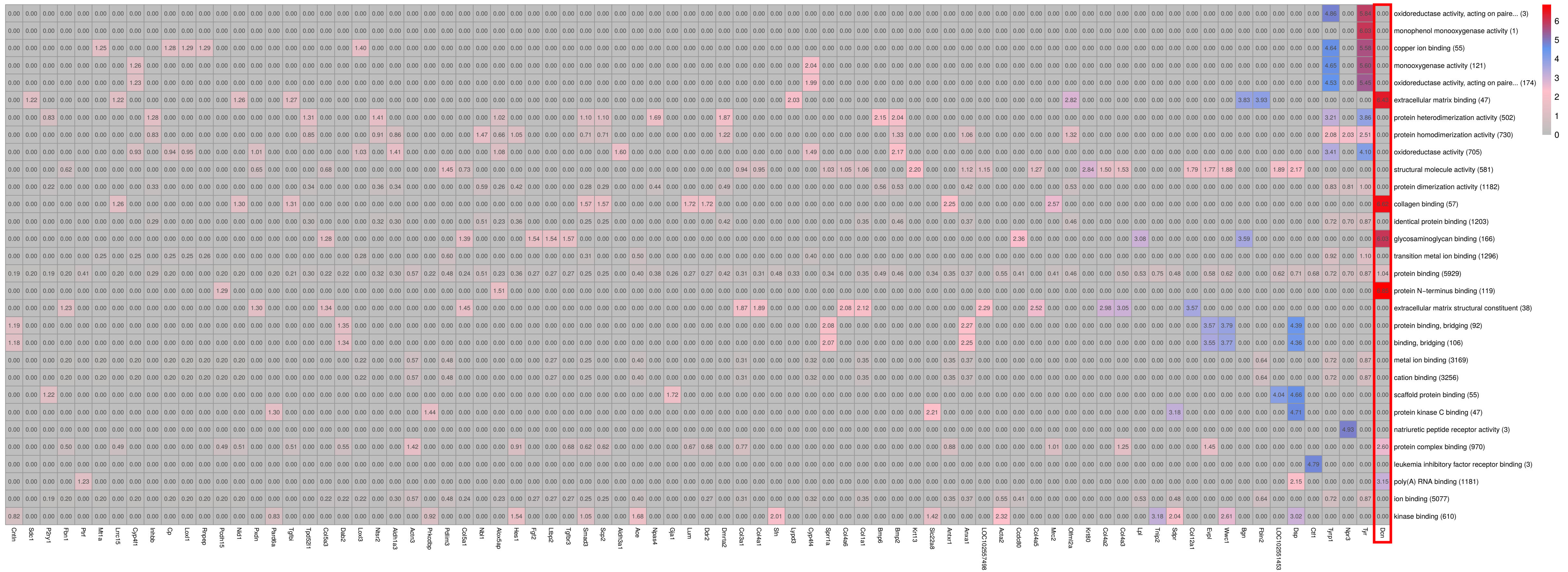

Supplement: Supplementary file 3 — Supplementary Data 1 [file 41467_2018_8125_MOESM3_ESM.pdf]
