# Supplementary material for: Mineralocorticoid receptor antagonism limits experimental choroidal neovascularization and structural changes associated with neovascular age-related macular degeneration
Source: Nat Commun. 2019 Jan 21;10:369. doi: 10.1038/s41467-018-08125-6 (PMC6341116; doi:10.1038/s41467-018-08125-6)
Supplement: Supplementary file 5 — Reporting Summary [file 41467_2018_8125_MOESM5_ESM.pdf]

natureresearch

Corresponding author(s): Francine Behar-Cohen

Reporting Summary

Nature Research wishes to improve the reproducibility of the work that we publish. This form provides structure for consistency and transparency in reporting. For further information on Nature Research policies, see Authors & Referees and the Editorial Policy Checklist.

Statistical parameters

When statistical analyses are reported, confirm that the following items are present in the relevant location (e.g. figure legend, table legend, main text, or Methods section).

n/a	Confirmed	
☐	☒	The exact sample size (n) for each experimental group/condition, given as a discrete number and unit of measurement
☐	☒	An indication of whether measurements were taken from distinct samples or whether the same sample was measured repeatedly
☐	☒	The statistical test(s) used AND whether they are one- or two-sided<br>*Only common tests should be described solely by name; describe more complex techniques in the Methods section.*
☐	☒	A description of all covariates tested
☐	☒	A description of any assumptions or corrections, such as tests of normality and adjustment for multiple comparisons
☐	☒	A full description of the statistics including central tendency (e.g. means) or other basic estimates (e.g. regression coefficient) AND variation (e.g. standard deviation) or associated estimates of uncertainty (e.g. confidence intervals)
☐	☒	For null hypothesis testing, the test statistic (e.g. F, t, r) with confidence intervals, effect sizes, degrees of freedom and P value noted<br>*Give P values as exact values whenever suitable.*
☒	☐	For Bayesian analysis, information on the choice of priors and Markov chain Monte Carlo settings
☒	☐	For hierarchical and complex designs, identification of the appropriate level for tests and full reporting of outcomes
☒	☐	Estimates of effect sizes (e.g. Cohen's d, Pearson's r), indicating how they were calculated
☐	☒	Clearly defined error bars<br>*State explicitly what error bars represent (e.g. SD, SE, CI)*

Our web collection on statistics for biologists may be useful.

Software and code

Policy information about availability of computer code

Data collection	RNAseq analysis using GOlandcape
Data analysis	The R code of GOlandscape and related documentation are available as open source at the web sites https://github.com/andreaprunotto/GOlandscape and https://zenodo.org/record/1474122. Essential parameters of GOlandscape include the numbers of displayed terms of four specific GO categories types: Biological Process, Cellular Component, Molecular Function and Pathways. These parameters, together with the number of genes appearing in the final heatmap, can be modified to deepen the GO analysis in each of these four directions. Various other plots are automatically produced by GOlandscape in order to support the analysis, namely a full representation of the step-wise procedure used to generate the final GO terms rank, a plot of the number of genes involved in each step of the analysis, and a plot of the GO rank as a function of the GO size.

For manuscripts utilizing custom algorithms or software that are central to the research but not yet described in published literature, software must be made available to editors/reviewers upon request. We strongly encourage code deposition in a community repository (e.g. GitHub). See the Nature Research guidelines for submitting code & software for further information.

Data

Policy information about availability of data

All manuscripts must include a data availability statement. This statement should provide the following information, where applicable:

- Accession codes, unique identifiers, or web links for publicly available datasets
- A list of figures that have associated raw data
- A description of any restrictions on data availability

All relevant data are available from the corresponding author upon reasonable request. RNA-sequencing data are available from ArrayExpress database at EMBL-EBI under accession number E-MTAB-7438 [https://www.ebi.ac.uk/arrayexpress/experiments/E-MTAB-7438/]. The images from figures are available at figshare [[https://figshare.com/articles/Antagonism_of_the_mineralocorticoid_pathway_limits_choroidal_neovascularization/7283648].

Field-specific reporting

Please select the best fit for your research. If you are not sure, read the appropriate sections before making your selection.

☒ Life sciences ☐ Behavioural & social sciences ☐ Ecological, evolutionary & environmental sciences

For a reference copy of the document with all sections, see nature.com/authors/policies/ReportingSummary-flat.pdf

Life sciences study design

All studies must disclose on these points even when the disclosure is negative.

Sample size	Sample size was calcullated for animal CNV study according to known variability of the model in rats and in mice and to the expected effect of the tested compounds and genetic manipulation as compared to the effects of known validated agents such as anti-VEGFs.
Data exclusions	We excluded CNV when traumatic laser-induced hemorrhage and fusion of lesions occurred. No other data were excluded
Replication	Replicability was tested by our statistical analysis between different experiments. With a linear mixed model, this allowed us to compare different experiments.
Randomization	Animals are randomized to treatment groups and within the same group rank of treatment is random<br>There was no control group in the clinical study.
Blinding	The laser model, treatment administration and imaging were performed by an individual, independent from the person who analyzed the data and who was unaware of the treatment groups. Statistical analysis was performed on raw data by a professional independent CRO.

Reporting for specific materials, systems and methods

Materials & experimental systems

n/a	Involved in the study	
☒	☐	Unique biological materials
☐	☒	Antibodies
☒	☐	Eukaryotic cell lines
☒	☐	Palaeontology
☐	☒	Animals and other organisms
☐	☒	Human research participants

Methods

n/a	Involved in the study	
☒	☐	ChIP-seq
☒	☐	Flow cytometry
☒	☐	MRI-based neuroimaging

Antibodies

Antibodies used	Mouse monoclonal anti-MR 6G1 (1:100, kindly provided by C. Gomez-Sanchez, Division of Endocrinology, University of Mississippi Medical Center, Jackson, MS)<br>FITC-GSL I-Isolectin B4 (FL-1201, 1:200, Vector, AbCys, Paris, France)<br>Rabbit anti-IBA1 antibody (019-19741, 1:400, Wako, Neuss, Germany)<br>Rabbit anti-DCN (ab175404, 1:1000, Abcam, Cambridge, UK)<br>Goat anti-Actin (sc1616, 1:2500, Santa Cruz Biotechnology, Heidelberg, Germany)
Validation	Already published by our group and by several other group in the field.

Methods employed herein are validated method to quantifiy CNV.
Negative controls were performed for all experiments.

Animals and other organisms

Policy information about studies involving animals; ARRIVE guidelines recommended for reporting animal research

Laboratory animals	Eight-week old male Long Evans rats from the Janvier Breeding Center (Le Genest-Saint-Isle, France).<br>Three-month old male mice with cell-type-specific MR deletion in endothelial, smooth muscle and myeloid cells, (i.e. Vecadh-MR-KO, Tie2-MR-KO, SMA-MR-KO and Lys-MR-KO mouse models, respectively), were generated in the C57BL/6 genetic background.<br>Floxed MR (MRf/f) mice67 (kindly provided by Dr. Berger, Heidelberg, Germany) were crossed with mice expressing an inducible Cre-ERT2 recombinase driven by the VE-Cadherin promoter (Cdh5(PAC)-CreERT2 line, kindly provided by Prof. Adams, London, UK68; to generate Vecadh-MR-KO mice) or by the -SMA promoter69 (kindly provided by Dr. Metzger, Strasbourg, France; to generating SMA-MR-KO mice).<br>Lys-MR-KO mice were obtained by mating mice expressing Cre recombinase in myeloid cells (LysMcre65; The Jackson Laboratory, USA) with the floxed MR mice. MRf/f littermates lacking the Cre transgene were used as controls.<br>Floxed MR mice were crossed with Tie2-Cre mice (strain: B6.Cg-Tg(Tek-cre)12Flv/J, The Jackson Laboratory) to obtain EC MR-/- (MRflox/flox//Tie2Cre) mice and corresponding MR f/f littermates as controls.<br>Animals were kept in pathogen-free conditions with food, water and litter and housed in a 12-hour light/12-hour dark cycle. Anesthesia was induced by intramuscular ketamine 40 mg/kg and xylazine 4 mg/kg in rats, and intraperitoneal injection of ketamine 50 mg/kg and xylazine10 mg/kg in mice. Animals were sacrificed by carbon dioxide inhalation or cervical dislocation.
Wild animals	*Provide details on animals observed in or captured in the field; report species, sex and age where possible. Describe how animals were caught and transported and what happened to captive animals after the study (if killed, explain why and describe method; if released, say where and when) OR state that the study did not involve wild animals.*
Field-collected samples	*For laboratory work with field-collected samples, describe all relevant parameters such as housing, maintenance, temperature, photoperiod and end-of-experiment protocol OR state that the study did not involve samples collected from the field.*

Human research participants

Policy information about studies involving human research participants

Population characteristics	Patients with refractory stable intraretinal or subretinal fluid present for ≥ 6 months in a row on monthly SD-OCT despite monthly intravitreal of the same anti-VEGF treatment (ranibizumab or aflibercept) were included. The total duration of anti-VEGF treatment before inclusion in the study was required to be ≥12 months to avoid the effect of improvement due to treatment initiation. The thickest macular A-scan from the inner limiting membrane to the RPE band was required to be ≥ 350 µm. Patients giving informed consent were included from September 2014 to March 2015.<br>Twenty patients with nAMD presenting with refractory intra- or subretinal fluid despite monthly intravitreal injections of anti-VEGF (≥12 months anti-VEGF treatment, ≥6 months refractoriness despite monthly injections, using the same anti-VEGF molecule (Aflibercept in 13 eyes / Ranibizumab in 8 eyes), ≥350 µm on thickest A-scan on optical coherence tomography (OCT)) consented to participate to a prospective pilot study. Refractoriness was defined as no reduction in exudative signs during the last 6 months<br>In 21 eyes of 20 patients with refractory nAMD (13 females, mean age 76.3 ± 7.7 (SD) years received 37.2± 17.1 (mean ± SD) anti-VEGF injections given over a mean period of 46.0 ± 19.8 (SD) months prior to study enrolment
Recruitment	Patients were monthly treated with anti-VEGF and were sequentially included according to inclusion citeria and acceptance to participate to the study.
